# How do young women approaching screening age interpret the NHS cervical screening leaflet? A mixed methods study of identifying interpretation difficulties, barriers, facilitators, and leaflet interpretation, engagement and future screening behaviour

**DOI:** 10.1080/21642850.2024.2361005

**Published:** 2024-05-30

**Authors:** Caroline Charlton, Angela M. Rodrigues

**Affiliations:** Department of Psychology, Northumbria University, Newcastle upon Tyne, UK

**Keywords:** Cervical cancer, cervical screening, health communication, informed decision making, risk communication

## Abstract

Background: Cervical cancer is a common cancer among young women aged 25–29 in England, and the NHS cervical screening leaflet is the first point of contact for those being invited for their first screening. This study aimed to explore how young women (18–24) understand and engage with the leaflet, as well as the barriers and facilitators associated with its interpretation, engagement, and screening intentions. Methods: The study used a mixed-methods approach, including a survey (*n* = 120) to identify interpretation difficulties and how they were affected by different characteristics, and a follow-up interview (*n* = 10) to assess the utility of the leaflet, identify issues with its practicality, and determine the factors that influence screening intentions. Results: The survey results showed that interpretation difficulties were common, particularly regarding HPV assessment, screening results, additional tests/treatment, and screening risks. Lower interpretation accuracy was associated with lower numeracy scores and non-white ethnicity. Despite these difficulties, participants had high confidence and motivation to engage with the leaflet. The interviews revealed knowledge gaps, issues with the leaflet's practicality, and a preference for digital information. Factors that were identified as barriers and facilitators of leaflet interpretation, engagement, and screening intentions included knowledge, social influence, beliefs about consequences, environmental context and resources, social role and identity, emotions and intentions. Conclusion: The current leaflet does not provide enough information for young women to make an informed decision about screening attendance. Implementing a digital invitation featuring simplified gist representation, targeted behaviour change techniques (BCTs), videos, and interactive tools can enhance education and promote screening behaviour. Future research should consider using digital tools and strategies to address existing barriers related to interpretation and engagement.

## Introduction

Within the UK, cervical cancer is the 14th most-common form of cancer diagnosis (Landy et al., 2016), however, in women considered of reproductive age (<35), it is the most-common form of cancer diagnosis (Salter, 2014). Human papillomavirus (HPV) is determined as the main causation of abnormal cervical cytology (precancers) and cervical cancer (Schiffman et al., 2018), with a UK causation rate of 80% (Mesher et al., 2015). Despite cervical cancer rates being halved between the late 1980s and the mid-2000s with the introduction of the National Health Services (NHS) cervical screening programme in 1988 (evidenced to significantly reduce cervical cancer incidence [Landy et al., 2020]), and the HPV vaccine in 2008 (with an observed 87% reduction in cervical cancer incidence among women vaccinated at ages 12–13 years old [Falcaro et al., 2021]), progress has notedly stagnated, with over a decade-long absence of progress (Cancer Research UK, 2020). The NHS cervical screening programme invites women aged between 25 and 49 to a cervical screening every 3 years and every 5 years for women aged between 50 and 64 (NHS, 2021), however recent figures show that less than 3-quarters (71.9%) of women who are invited uptake with the service, this figure further decreases within women aged between 25 and 29 (61.9%), and within more deprived areas (NHS, 2019). This lack of engagement may explain the sharp incline of cervical cancer rates (54%) across the last decade, within 25–29-year-olds (Cancer Research UK, 2020), and suggests that cervical screening as an intervention could be further optimised.

The NHS cervical screening leaflet is provided to women as part of the invitation to the screening programme. The leaflet serves as a health communication tool, aiming to raise awareness of cervical screening and facilitate informed decision-making. It provides essential information about cervical cancer, the screening procedure, potential results, and conveys associated risks and benefits of screening (NHS, 2021).

Previous research on non-engagement with the cervical screening programme has predominantly focused on identifying barriers to uptake. These barriers include practical factors such as inconvenient appointment times and lack of transportation (Verdoodt et al., 2015), psychosocial factors such as fear of cancer or procedure-related pain (Patel et al., 2018), and socio-economic inequalities (Douglas et al., 2016). Specific barriers among younger women (aged 25–29) include embarrassment (Jo’s Cervical Cancer Trust, 2019), body confidence issues (Jo’s Cervical Cancer Trust, 2018), and limited knowledge about cervical screening (Jo’s Cervical Cancer Trust, 2017). Concerns have been raised regarding HPV vaccine engagement, low perception of cervical cancer risk, and social influence contributing to declining cervical screening uptake (Sadler et al., 2013). Understanding how health communications contribute to this downward trend is crucial for the cervical screening programme as a whole.

Limited research exists on the interpretation and understanding of cervical screening communications, with only one study examining interpretation of the 2017 version of the NHS leaflet in relation to recipients’ characteristics such as educational attainment, numeracy, and ethnicity (Okan et al., 2019). Results revealed interpretation difficulties and knowledge gaps among women of eligible screening age, particularly lower educational attainment, lower numeracy skills, and non-white ethnic backgrounds. However, the study had limitations as it focused mainly on attendees and lacked non-attenders perspectives. Limited representation of first-time invitees may have influenced interpretation of the leaflet due to prior screening experience.

Considering the transition to HPV-primary testing and the circulation of an updated cervical screening leaflet, it is necessary to assess the effectiveness of the leaflet in conveying information to women approaching screening eligibility. Since the invitation letter's accompanying leaflet serves as the initial point of contact for young women, it is important to explore its suitability in providing knowledge and fostering positive intentions for engaging in the screening programme. Additionally, it is crucial to investigate leaflet perception in terms of utility and how young women engage with it upon invitation, as limited engagement with screening health communications contributes to low uptake of cancer screening (Ghanouni et al., 2017). Notably, previous research has not explored barriers or facilitators of interpretation or engagement with the cervical screening leaflet among individuals with different characteristics.

This exploratory research aims to develop a comprehensive understanding of young women's interpretation of the cervical screening leaflet and identify factors hindering utilisation and engagement with the leaflet and screening programme. By gaining in-depth insights into young women approaching cervical screening age’s (18–24 years old) attitudes and perceptions of the leaflet and service upon first invitation, it is hoped that improvements that address current low engagement within the cervical screening programme among young women in North East England can be made (Office for Health Improvement and Disparities, 2023). The research questions are:

Phase A:

1. Do young women experience interpretation difficulties with the current cervical screening leaflet, and do previously identified difficulties persist?
2. How do individual characteristics, including socio-demographics, cancer-risk perception, prior HPV vaccine engagement, and numeracy, impact young women's accuracy, confidence in interpretation, and motivation for future screening behaviour?

Phase B:

1. How do young women perceive, utilise, and regard the cervical screening leaflet as their initial source of information about the screening programme?
2. What are the primary perceived barriers and facilitators regarding interpretation, engagement, and future screening behaviour relating to participant characteristics?

## Methods

### Design

This study employed a sequential mixed-methods design. This design was chosen to aid recruitment efforts due to COVID-19 restrictions.

### Participant information

#### Phase A

One hundred and fifty-six participants were recruited through purposeful and convenience sampling via social media. Due to the restrictions imposed by the first COVID-19 lockdown in the UK, using social media platform allowed for a swift dissemination of the survey, enabling access to potential participants. Eligible participants were women aged 18–24 residing in North East England who had not undergone cervical screening or been diagnosed with cervical cancer. Exclusions included individuals with prior cervical cancer diagnosis or screening to minimise interpretation biases (Okan et al., 2019). Interested individuals were contacted to provide study details and assess eligibility, with eligible participants receiving an anonymous survey link after confirmation.

#### Phase B

Out of 156 survey participants, 28 expressed interest in participating in the optional qualitative phase. The researcher contacted these participants to provide additional information. Considering COVID-19 restrictions, flexible options e.g. online video calls, social media platforms, and telephone appointments were offered to accommodate the participants’ preferences and comfort.

### Materials

#### Phase A – online survey

##### Leaflet

The study incorporated the latest publication of the cervical screening leaflet titled ‘Cervical Screening: Helping you Decide’ (February 2020 version; Cervical screening: leaflet for women considering screening – GOV.UK (www.gov.uk).

##### Survey items

Survey design and items were replicated from Okan et al. (Okan et al., 2019) (adapted where necessary to reflect the leaflet used) and followed their survey structure; sociodemographic profile and cognitions, leaflet interpretations and confidence, and motivation to engage with cervical screening service (supplementary file 1: Table 1).

##### Participant characteristics and cognitions

Sociodemographic variables included age, education level, ethnic and religious profiles (adopted from 2011 Census programme) (2011 Census Programme, 2009a; 2011 Census Programme, 2009b), employment status (to calculate social grade, developed from the National Readership Survey [NRS]) (National Readership Survey, 2021), first language, receipt of invitation to the cervical screening programme, and knowledge of someone with a cervical cancer diagnosis (adopted from Okan and colleagues) (Okan et al., 2019).

The survey included items related to behaviour and cognitions, e.g. HPV vaccine engagement (multiple choice and open responses), cancer risk perception (measured via 5-point Likert scale), and numeracy. Numeracy was assessed using a validated 3-item assessment that included open-ended questions (Schwartz et al., 1997). Each correct answer was scored on a 0–3 scale, scores below 3 indicating low numeracy. The inclusion of this numeracy assessment aimed to examine its impact as a barrier to accessing healthcare, including cancer screening services (Kobayashi et al., 2014; Oldach & Katz, 2014), and its influence on informed decision making within health communications (Smith et al., 2016).

##### Interpretation

Items assessing participant interpretation were adapted from Okan et al. (2019) which underwent three rounds of cognitive interviews to ensure accurate interpretation and minimise reading barriers. The researcher developed three additional items, including screening risk to pregnancy, HPV vaccine protection, and result and sample storage questions. All newly developed items were pretested with five participants to confirm understanding. The final set of items included 19 true/false items and two open-response items (Supplementary File 1, Table 1). Participants rated their confidence in their interpretation on a 6-point scale ranging from 50%−100% (just guessing – absolutely certain). The true/false format was chosen for ease of use and ability to detect partial or mixed misunderstandings (Couch et al., 2018; de Bruin & Fischhoff, 2000; Okan et al., 2019).

##### Motivation to engage

A single item was developed to measure participants’ motivation to engage with the cervical screening programme in the future. The item's phrasing was informed by the refined 14-domain Theoretical Domains Framework (TDF) (Cane et al., 2012) to ensure accurate interpretation of participants’ motivation.

#### Phase B

##### Leaflet

All sections of the leaflet mentioned above were incorporated to explore interpretation difficulties.

##### Interview schedule

One-to-one interviews were conducted using a semi-structured interview schedule (supplementary file 1, Table 2), defining the behavioural category of interest through the TACT principle (Target, Action, Context, and Time) (Ajzen, 2006). The behaviour under consideration was young women's (18–24) interpretation of the cervical screening information leaflet, engagement, and future screening behaviour after reading the leaflet. The interview schedule comprised three sections: leaflet utility (pre/post leaflet interaction), leaflet interpretation (think-aloud procedure), and identifying barriers and facilitators to leaflet engagement and future screening behaviour. The interview schedule underwent piloting with two participants (not in the final sample) to ensure understanding of items and to verify if it elicited relevant data to address the research questions.

##### Interpretation: think-aloud protocol

For evaluation of leaflet interpretation, participants were asked to ‘think aloud’ as they read through the leaflet. This process involves verbalisation of thoughts of which are usually silent, whilst participants are completing a primary task (Johnstone et al., 2006). Think aloud protocols are an important procedure within which verbalisations enable access to participants cognitive processes due to the representation of the short-term memory contents at the point in time during task engagement (Simon & Ericsson, 1984). For full protocol, and materials used within, see supplementary file 2.

### Procedure

This research was provided ethical approval by the psychology department ethics committee at Northumbria University (Ref: 23410).

#### Phase A

Participants were assessed for eligibility and provided with a Qualtrics link. They first viewed an embedded information sheet outlining the study's purpose, requirements, and eligibility criteria. Participants provided informed consent and completed the survey items on socio-demographics, interpretation, confidence of interpretation, and motivation to engage. At survey completion, interested participants left an email address for phase B, and were given a debrief sheet with information on the research purpose, data request, and right to withdraw. The survey took approximately 15 min to complete.

#### Phase B

One-to-one interviews were conducted by an experienced researcher unfamiliar to participants, with email communication and introductions at 4 weeks post phase A to prevent interpretation bias. Informed consent was obtained electronically pre-interview. On the interview day, participants received the cognitive think-aloud task instructions, a practice leaflet, and the cervical screening leaflet via email. The think-aloud protocol was explained, participants were encouraged to seek clarification, and their right to withdraw was emphasised. The interview followed the schedule, with breaks pre – and post-cognitive think-aloud task. After the interview, participants were debriefed, informed about data handling, how to request results, and reminded of their withdrawal right. Any queries were addressed. The interviews, lasting 40–120 min, were fully anonymised.

### Analysis strategy

#### Phase A

Overall accuracy of interpretation scores for each participant were calculated by totalling the number correct responses to interpretation items, with missing items coded as incorrect. Data was then analysed via multiple linear regressions, followed by hierarchal regressions to determine the variation in variance between demographical and cognitive characteristics upon leaflet interpretation accuracy, mean confidence, and motivation to engage. Further analysis of individual interpretation and confidence items were conducted to determine whether specific areas of the leaflet are causing interpretation problems. Correlational analyses were conducted to examine the relationship between interpretation accuracy, confidence of accuracy and motivation to engage. Finally, A chi square analysis was conducted to examine the association between cancer risk perception and HPV vaccine engagement. For coding and analysis strategy, see supplementary file 3.

#### Phase B

Interviews were audio-recorded and transcribed verbatim. Transcripts were analysed and coded by one researcher using the thematic analysis 6-stage approach (Braun & Clarke, 2006); (1) data familiarisation, (2) generating codes close with the dataset (3) theme searching, (4) theme reviewing, (5) naming and identifying themes and finally (6) producing a written report of the analysis. Codes generated identifying facilitators or barriers of leaflet interpretation, engagement, or future screening behaviour, were mapped to TDF domains. Codes linked to more than one TDF domain were mapped to the domain with most relevance. Transcripts were reviewed by participants to ascertain the accuracy in capturing their responses, and whether resultant themes adequately reflected the behaviour being assessed (see supplementary file 4 for further detail on transcript analysis and theme development).

## Results

### Phase A

#### Participant characteristics

The survey initially attracted 156 participants, with 7 excluded for not meeting age criteria and 29 removed for non-completion or completing the survey under 5 min. The decision to exclude participants completing the survey within 5 min ensured an attentive sample, as this timeframe was half the average completion time within questionnaire pilots. The final sample (*n* = 120), mean age of 22.50 (SD = 1.461), comprised 65% participants of white ethnicity, 87.5% higher education level, and 63.3% no religious identity. See supplementary file 1, Tables 3 and 4, for detailed participant characteristics in both phases, and comparative statistics between the recruited participant sample and population data.

#### Leaflet interpretations

Participants correctly answered a mean average of 13.98 (SD = 3.324; Range = 7–20) out of 21 items assessing leaflet interpretations, suggesting common interpretation difficulties. Regression results revealed the strongest predictor of interpretation accuracy was numeracy, followed by ethnicity, (see Table 1). Accuracy of interpretation was lower in participants who scored lower on numeracy items and participants from non-white ethnic backgrounds. Further hierarchal regression results identified that only ethnicity (block 1) contributed 19.3% of variance to interpretation scores, and only numeracy (block 2) contributed an additional 13.2% of variance to interpretation accuracy scores (see Table 2).

**Table 1.** Multiple linear regression models predicting interpretation accuracy, mean reported confidence and motivation to engage.

	Interpretation Accuracy (0–21)			Mean reported confidence (50–100)			Motivation to engage (1–5)		
	*B* (SE)	*β*	*P*	*B* (SE)	*β*	*P*	*B* (SE)	*β*	*P*
Education (1 = higher; 0 = other)	.731 (.819)	.073	.374	2.326 (3.211)	.063	.470	.452 (.368)	.179	.053
Ethnicity (1 = white; 0 = other)	2.112 (.610)	.304	.001	4.063 (2.391)	.159	.092	239 (.172)	.136	.169
Religion (1 = religious; 0 = non-religious)	−.921 (.596)	−.134	.125	-.795 (2.336)	-.031	.734	-.065 (.168)	-.037	.701
Social Grade (1 = higher (C1, B, A); 0 = lower (C2, D, E)	−.374 (.613)	−.050	.543	-.984 (2.404)	-.036	.683	322 (.173)	.171	.066
First Language (1 = English; 0 = other)	.579 (.640)	.082	.368	4.275 (2.511)	.165	.091	.073 (.181)	.041	.686
HPV Vaccine Engagement (1 = yes; 0 = no)	−.382 (.570)	−.057	.503	−3.983 (2.234)	-.162	.077	.203 (.161)	.121	.209
Perceived Cancer Risk (1–5)	−.275 (.291)	−.075	.347	.321 (1.140)	.024	.779	.169 (.082)	.181	.042
Numeracy (0–3)	1.205 (.270)	.373	.000	4.723 (1.059)	.397	.000	-.020 (.076)	-.025	.790
Model Statistics	*R*^2 ^= .325			*R*^2 ^= .234			*R*^2^ = .152		
	*F*(8, 111) = 6.679, *P* < .001			*F*(8, 111) = 4.241, *P* < .001			*F*(8, 111) = 2.484, *P* = .016		

*Social Grade Categories: A = Higher managerial, administrative, and professional; B = Intermediate managerial, administrative and professional; C1 = Supervisory, clerical and junior managerial, administrative and professional; C2 = Skilled manual workers; D = Semi-skilled and unskilled manual workers; E = State pensioners, casual and lowest grade workers, unemployed with state benefits only. HPV = Human papillomavirus.

**Table 2.** Hierarchal linear regression models predicting interpretation accuracy, mean reported confidence and motivation to engage.

	Interpretation accuracy (0–21)			Mean reported confidence (50–100)			Motivation to engage (1–5)		
	*B* (SE)	*β*	*P*	*B* (SE)	*β*	*P*	*B* (SE)	*β*	*P*
**Block 1**	.								
Education (1 = higher; 0 = other)	.780 (.879)	.078	.377	2.214 (3.432)	.060	.520	.481 (.234)	.191	.042
Ethnicity (1 = white; 0 = other)	2.011 (.654)	.290	.003	4.043 (2.550)	.158	.116	.228	.130	.192
Religion (1 = religious; 0 = non-religious)	−1.243 (.630)	−.181	.051	−1.515 (2.459)	−.060	.539	−.083 (.167)	−.048	.619
Social Grade (1 = higher (C1, B, A); 0 = lower (C2, D, E)	.059 (.648)	.008	.928	1.017 (2.530)	.037	.688	.307 (.172)	.163	.077
First Language (1 = English; 0 = other)	.739 (.672)	.105	.274	4.566 (2.624)	.177	.084	.150	.085	.403
Model statistics	*R*^2^ = .193			*R*^2^ = .094			*R*^2 ^= .104		
	*F*(5, 114) = 5.453, *P* < .001			*F*(5, 114) = 2.370, *P* = .044			*F*(5, 114) = 2.640, *P *= .027		
**Block 2**									
Education (1 = higher; 0 = other)	.731 (.819)	.073	.374	2.326 (3.211)	.063	.470	.452 (.231)	.179	.053
Ethnicity (1 = white; 0 = other)	2.112 (.610)	.304	.001	4.063 (2.391)	.159	.092	.239 (.172)	.136	.169
Religion (1 = religious; 0 = non-religious)	−.921 (.596)	−.134	.125	−.795 (2.336)	−.031	.734	−.065 (.168)	−.037	.701
Social Grade (1 = higher (C1, B, A); 0 = lower (C2, D, E)	−.374 (.613)	−.050	.543	−.984 (2.404)	−.036	.683	.322 (.173)	.171	.066
First Language (1 = English; 0 = other)	.579 (.640)	.082	.368	4.275 (2.511)	.165	.091	.073 (.181)	.041	.686
HPV Vaccine Engagement (1 = yes; 0 = no)	−.382 (.570)	−.057	.503	−3.983 (2.234)	−.162	.077	.203 (.161)	.121	.209
Perceived Cancer Risk (1–5)	−.275 (.291)	−.075	.347	.321 (1.140)	.024	.779	.169 (.082)	.181	.042
Numeracy (0–3)	1.205 (.270)	.373	.000	4.723 (1.059)	.397	.000	−.0.20 (.076)	−.025	.790
Model Statistics	*R*^2 ^= .325; Δ*R*^2 ^= .132 (13.2%)			*R*^2 ^= .234; Δ*R*^2 ^= .140 (14%)			*R*^2 ^= .152; Δ*R*^2 ^= .048 (4.8%)		
	*F*(8, 111) = 6.679, *P *< .001			*F*(8, 111) = 4.241, *P *< .001			*F*(8, 111) = 2.484, *P* = .016		

*Social Grade Categories: A = Higher managerial, administrative and professional; B = Intermediate managerial, administrative and professional; C1 = Supervisory, clerical and junior managerial, administrative and professional; C2 = Skilled manual workers; D = Semi-skilled and unskilled manual workers; E = State pensioners, casual and lowest grade workers, unemployed with state benefits only. HPV = Human papillomavirus.

Further analyses of individual interpretation items found that participant performance was distinctly poorer within items assessing additional tests and treatment, screening results, screening risks, and HPV. Although performance was higher within colposcopy and main goal of screening and benefits items, there were still common misinterpretations (see Table 3 for % interpretation accuracy for all items). Further analysis of how participant estimates within screening results items were distributed determine there is underestimation of adverse screening results (supplementary file 1: Tables 7 and 8).

**Table 3.** Survey results of accuracy and confidence for interpretation assessment Items.

Item	% Correct	Confidence (Mean)
**Main goal of cervical screening and screening benefits**		
Cervical screening prevents as many as 70% of cervical cancer deaths each year in the UK. (T)	90.8%	84.92
Cervical screening is mandatory. (F)	85.0%	89.00
The main goal of cervical screening is to find cancer that is already there. (F)	82.5%	88.67
Cervical screening lowers the risk of getting cervical cancer. (T)	91.7%	89.75
**HPV**		
HPV is a sexually transmitted infection. (STI) (T)	67.5%	84.42
HPV can be passed on during sexual intercourse. (T)	91.7%	90.67
Men can’t get HPV. (F)	82.5%	82.58
HPV usually doesn’t need any treatment. (T)	58.3%	81.42
Using condoms lowers the risk of getting HPV. (T)	83.3%	86.42
The HPV vaccine protects against all types of high-risk HPV which can lead to cervical cancer. (F)	49.2%	83.75
**Additional tests and treatment**		
An HPV negative test result rules out the possibility that there are any abnormal cells. (F)	46.7%	81.33
A woman who does not have abnormal cells could get an abnormal test result. (T)	75.8%	79.58
If a woman is HPV negative but has potentially abnormal cells, she is referred for further testing. (F)	52.5%	84.92
You are referred for a colposcopy if you test positive for HPV. (F)	40.0%	84.08
All cervical screening samples are tested for abnormal cells. (F)	36.7%	80.92
**Screening results**		
Imagine 1000 women who have attended a cervical screening. About how many of them will … .		
Have an HPV positive result? (130)	71.7%	83.67
Have cells that could be cancer? (40)	59.2%	72.00
All previous screening results are stored on a secure computer system for a minimum of 10 years. (F)	26.7%	88.17
**Colposcopy**		
A colposcopy checks if there are abnormal cells in the cervix. (T)	80.0	83.92
**Screening risks**		
Imagine a woman has had a cervical screening test. If she gets pregnant later, it is slightly more likely that her baby will be born early due to her having a cervical screening test previously? (F)	65.8%	84.75
Cervical screening can lead to treatment of abnormal cells that is not needed. (T)	60.0%	76.92

*T = True; F = False. HPV = HPV = Human papillomavirus. Correct leaflet interpretations (based on information leaflet) in brackets.

#### Reported confidence

Despite participants’ interpretation difficulties, their self-reported confidence was relatively high, averaging 83.89 (SD = 12.24), with scores ranging from 52.38% to 100%. Mean confidence scores were moderately correlated with overall accuracy across all interpretation items (*r* = .437, *P* < .001), indicating participants tended to be more confident when demonstrating a better understanding of the interpretation items (supplementary file 1: Table 5). Regression results identified numeracy as the sole significant predictor of mean self-reported confidence, with higher confidence in participants scoring higher in numeracy items (see Table 1). Further hierarchical regression results showed that none of the socio-demographic variables (block 1) significantly predicted mean reported confidence scores but collectively accounted for 9.4% of the variance. Only numeracy (block 2) significantly contributed to mean confidence scores, explaining an additional 14% of the variance (Table 2). However, despite significant, the relationship between the variables was weak.

#### Motivation to engage

Although participants faced interpretation difficulties with various leaflet items, they expressed high perceived importance of attending a cervical screening appointment after reading surveyed leaflet sections (mean = 4.56, SD = .838), with importance rated on a scale from 1 (low) to 5 (high) importance. Participants’ motivation to engage with future cervical screening was weakly but significantly correlated with self-reported confidence (r = .229, *P* = .012), suggesting greater confidence in interpreting the leaflet was associated with higher likelihood of attending a cervical screening appointment (supplementary file 1: Table 5). Regression results indicated that the sole significant predictor of motivation to engage was perceived cancer risk, with higher motivation observed in participants perceiving a higher cancer risk (see Table 1). Further hierarchical regression results revealed only education (block 1) significantly predicted motivation to engage, explaining 10.4% of the variance. Perceived cancer risk emerged as a significant predictor, contributing an additional 4.8% of the variance, and education no longer predicted motivation to engage once cognitive variables were incorporated (Table 2). It is crucial to note that regression assumptions were violated in both models, raising concerns about the reliability of the results.

Most participants perceived their cervical cancer risk as between low and neutral (*n* = 103). Reasons stated for lower perception of cancer risk (neutral – very low risk) were, they had the HPV vaccine, no family history, being too young to develop cervical cancer, and leading a healthy lifestyle. Further chi-square analyses revealed that although slightly more participants who had the HPV vaccine were reporting lower cancer risk perception, the difference was not significant *X*^2^(4, *N* = 120) = 4.72, *P* = .317, suggesting the overall trend of lower cancer risk perception is not related to previous HPV vaccine engagement. For frequency of participants reporting low, neutral or high perceived cancer risk as a result of HPV vaccine engagement see supplementary file 1: Table 6.

### Phase B

#### Participant characteristics

Contact details from 28 participants were given at the end of the first survey expressing interest in phase B of the study and were promptly contacted. 15 people replied back initially, 3 dropped out before interviews could be organised and 2 had scheduling issues that could not be resolved. The final sample (*n *= 10), with a mean age of 22.90 (SD = 1.524), included 90% participants of white ethnicity, 80% of higher education level, and 70% no religious identity. Despite initial plans to recruit a minimum of 13 participants as per recommended practice (Francis et al., 2010), due to difficulty recruiting during COVID-19 restrictions in early 2020, despite several efforts, data collection ceased at 10 participants.

#### Leaflet interpretations

Within this section of the interview, five themes were identified; two of which reflected the participants difficulty in interpreting the information from the leaflet (confusion and misinterpretation of the purpose of screening and gaps in knowledge [HPV, results and further treatment, screening risks]), two of which address positive leaflet reactions (positive reaction to diagrams and statistical information and screening procedure section detailed and informative), and 1 which reflects upon negative leaflet reactions (concerns regarding pain, age of eligibility, and screening risks). See Table 4 for relevant quotes.

**Table 4.** Identified themes in cognitive think-aloud section and illustrative quotes.

Theme	Sub-theme(s)	Illustrative quotes	Relevant leaflet quotes (sections)
Confusion and Misinterpretation of the purpose of screening	-	‘It’s funny because cervical screening doesn’t seem like it would prevent cervical cancer, it seems like more of a thing you would have when going through diagnosis of cervical cancer instead … .because … .well my sister said that it looks for cancerous cells so you get them removed before it develops into something a lot more serious anyways, so I’m not sure what they mean by preventative unless they mean like … well … preventing it from getting any worse, or catching it in super early stages’. (P1)	‘NHS cervical screening helps prevent cervical cancer.’ (Page 2)
Gaps in Knowledge	HPV	‘So … I thought HPV was an STI but I’m a little unsure as it doesn't really say that it just says close skin to skin contact, like to me that feels like an STI, but because they aren't saying it outright I’m doubting myself.’ (P1)	‘HPV is very common. Most people will get the virus at some point in their life It is spread through close skin to skin contact during any type of sexual activity with a man or woman.’ (Page 4)
	Results and further treatment	‘I would like to know how often a false positive or false negative actually happens, that kind of thing is pretty important to know when making decisions about optional procedures like screening’. (P9)	‘No screening test is 100% effective. In cervical screening this is because: an HPV infection or abnormal cells can sometimes be missed (a ‘false negative’ result). Abnormal cells can develop and turn into cancer in between screening tests. There is a small chance that a result says abnormal cells are found when the cervix is normal (a ‘false positive’ result). If screening does not find abnormal cells this does not guarantee that you do not have them, or that they will never develop in the future.’ (Page 6)
	Screening risks	‘I think they need to give us a better idea of numbers here. I mean 16 out of 100 women is huge to me in terms of risk, but like how often are women having their babies being born early each year or something? Like maybe an updated statistic?’ (P2)	‘This may affect around 16% of women (16 in 100) who have this more extensive treatment and then have a baby.’ (Page 12)
Positive Reaction to Diagrams and Statistical Information	-	‘The statistics show the importance of attending and is generally positive, as in if you attend you drastically improve your chances of preventing a cervical cancer death.’ (P10)	‘In England, cervical screening currently prevents 70% of cervical cancer deaths. If everyone attended screening regularly, 83% could be prevented.’ (Page 2)
Screening Procedure Section Detailed and Informative	-	‘I think it’s really helpful that they informed us that it is possible to ensure it is a female nurse as that will make a lot of people feel more comfortable when making an appointment.’ (P9)	‘Cervical screening us usually carried out by a female nurse of doctor. If you want to make sure a woman carries out your test, you can ask for this when you make your appointment.’ (Page 6)
Concerns	Screening exam and results	‘The idea of screening being painful does worry me, it’s the one thing I think that makes me think again about going, I have a friend who said it made her cry it was that bad! So, it makes me a bit nervous thinking that my turn is coming up soon.’ (P7)	‘You might feel some discomfort, but this should go away quickly. If it feels painful, tell the nurse or doctor and they will try to make it more comfortable for you.’ (Page 7)
	Screening eligibility	‘But then I’m sure there have been quite a few news articles on women younger than 25 like dying of cervical cancer like … what’s her name again … the big brother star … oh Jade Goody, didn’t she die from that? I’m sure she did … and she was so young!’ (P5)	‘We offer screening every 3 years from age 25–49 and every 5 years from 50 to 64.’ (Page 2)
	Screening risks	‘Well … that’s certainly a lot more risks than benefits, look at the size of that paragraph!’ (P1)	See section 11 (page 12)

*For relevant leaflet sections please see (February 2020 version; Cervical screening: leaflet for women considering screening – GOV.UK (www.gov.uk).

#### Leaflet utility

Within this section of the interview, three themes were identified; two of which represented barriers within leaflet utility; (1) Leaflet not Practical (ease of use, time for engagement, and suitability of leaflet to provide information regarding intimate procedure)and (2) Leaflet not Appealing (non-engaging format, jumbled layout, and use of clinical terminology off-putting) and 1 which represented participant format preference for Digital Information (Format practical for younger women, more engaging, relaxed informal format, eases environmental concerns). See Table 5.

**Table 5.** Leaflet utility themes and illustrative quotes.

TDF domain/theme	Sub-theme(s)	Barrier/facilitator/mix	Illustrative quotes
Leaflet not Practical (*Environmental Context & Resources*)	–	Barrier	‘Well with the physical leaflet, like I said before I would most likely just forget all about it and wouldn't be able to find it once I wanted to read it.’ (P1)
Leaflet not Appealing (*Environmental Context & Resources*)	–	Barrier	‘Not really. It could be a lot more attractive with some brighter colours or more diagrams and things like that.’ (P8)
Preference for Digital Format	–	Facilitator	‘I would probably have to say something like this online version I am using today. Since it can be accessed by my phone and stuff that just makes it easier for me since I practically live on my phone.’ (P2)

#### Barriers and facilitators of leaflet interpretation, engagement and future screening behaviour

Seven main themes were identified as barriers and facilitators reflecting leaflet interpretation, engagement and future screening behaviour: (1) knowledge (lack of knowledge [barrier], prior knowledge [barrier], confidence of knowledge [mixed], (2) social influences (friend/family support [facilitator], friend/family prior experience [mixed], social influence [mixed], (3) beliefs about consequences (HPV vaccine beliefs [barrier], low perception of cancer risk [barrier]), (4) environmental context and resources (education [barrier]), (5) social role and identity (religion and cultural beliefs [barrier]), (6) emotions (fear of regretting non-attendance [facilitator], fear/anxiety of pain/procedure [barrier], fear of cancer [barrier], embarrassment – body image, intimate procedure, reluctance to discuss with healthcare professional [barrier] and (7) intentions (ambivalence of Screening Importance [barrier]) (See Table 6).

**Table 6.** Barriers and facilitators of leaflet interpretation, engagement and future screening behaviour themes and illustrative quotes.

TDF domain/theme	Sub-theme(s)	Barrier/facilitator/mix	Illustrative quotes
Knowledge	Lack of knowledge	Barrier	‘I am uncertain, it does seem important, yes, it is important, but I think I need to look more into this as the leaflet doesn’t tell me much about what I want to know, before making a concrete decision.’ (P7)
	Prior knowledge	Barrier	‘It was helpful to know that screening is actually something that prevents cervical cancer as I did not know that I’m pretty sure my friends that have been said they look for cancerous cells, so I don’t know. But it’s good to know the actual purpose of the test.’ (P5)
	Confidence of knowledge	Mix	‘I think the leaflet has given me a pretty solid idea of what to expect during a screening and what results I could get etc. So, yes, I’m quite happy with what I know now because of the leaflet.’ (P6)
Social Influences	–	Mix	‘Like I would of just asked my mum about it since she has been for screening before, as I think she can give me more information than a leaflet can.’ (P3)
Beliefs about consequences	HPV vaccine beliefs	Barrier	‘Knowing that I’m protected against pretty much all the types of HPV which cause cervical cancer just makes me feel a lot more relaxed about it.’ (P3)
	Low perception of cancer risk	Barrier	‘It seems that the chances of HPV turning into cancer are really low, so the cancer is a pretty rare thing to happen as a result.’ (P2)
Environmental context and resources	Education	Barrier	‘Possibly there could be an issue with understanding the proportionate level of risk due to the colposcopy procedure if they aren’t very numerate? Which would raise problems with engaging with the programme.’ (P10)
Social/Professional role and identity	Religion and culture	Barrier	‘ … however, it failed to include anything for religious individuals or people from different cultural backgrounds. Are they allowed screening, or is I recommended? Would they have their own ways of performing the screening test? I’m not sure but I think maybe a link for further information could have been included for those who need it.’ (P9)
Emotions	–	Barrier	‘The idea of screening being painful does worry me, it’s the one thing I think that makes me think again about going, I have a friend who said it made her cry it was that bad! So, it makes me a bit nervous thinking that my turn is coming up soon.’ (P7)
Intentions	Ambivalence of screening importance	Barrier	‘Well like I said earlier, this is something that I will definitely be discussing with my mum, but overall, my feeling are right now that it’s not a major priority.’ (P3)

## Discussion

### Summary of key findings

The current NHS cervical screening leaflet doesn't meet the informational needs of those approaching screening age. Numerous interpretation difficulties and knowledge gaps persist in key areas, such as screening purpose, HPV, results, further tests and treatment, and screening risks, echoing prior research (Chorley et al., 2018; Marlow et al., 2020; Okan et al., 2019; van der Meij et al., 2019, july 1). Despite misinterpretations, young women express high confidence in their understanding, indicating overconfidence in their acquired knowledge, as previously identified (de Bruin et al., 2007; Okan et al., 2019; Olsson, 2014). Interestingly, phase B participants show contrasting results, with low confidence in leaflet knowledge. This disparity may stem from guessing in phase A and reluctance to admit uncertainty (Okan et al., 2019). However, confidence strongly influences future screening behaviour motivation in both phases, as observed previously (Kelly et al., 2016; Lucero & Chen, 2020). Improving young women's cervical screening knowledge is crucial for boosting engagement. Yet, findings suggest disparities in interpretation accuracy among those from non-white backgrounds or with lower numeracy skills, underscoring the challenge of creating inclusive health communication materials.

Health literacy encompasses an individual's competency, knowledge, and motivation to comprehend and utilise health communications for decision-making (Wittink & Oosterhaven, 2018). This aligns with previous research indicating low numeracy skills impede accurate interpretation of health communications, particularly, their ability to ascertain and act upon risk information within health communications (Okan et al., 2019; Petrova et al., 2018; Smith et al., 2015). Despite experiencing difficulties in interpreting numerical information, participants in phase B positively evaluated numerical data in the leaflet. Evidence supports the preference for numerical inclusions in health communications, deemed more reliable than verbal descriptions when conveying health risks (Gurmankin et al., 2004; Jenkins et al., 2019; Visschers et al., 2009). Simple visual aids are favoured by diverse audiences (Garcia-Retamero & Cokely, 2017; Gigerenzer & Edwards, 2003). However, the findings suggests that further simplification or alternative formats of numerical information may enhance understanding of the leaflet.

Evidence supports the effectiveness of a ‘less is more’ approach in health communication and informed decision-making. Fuzzy trace theory (Reyna, 2008), a dual processing model, posits that health information processing involves both verbatim (precise) and gist (vague meaning) representations. Within complex information (e.g. risk information), the ability to extract the gist representation is influenced by literacy and numeracy (Reyna, 2008; Smith et al., 2015). Developing health information based on gist representation has demonstrated improved knowledge, comprehension, and decision-making across various health domains, such as colorectal screening (Smith et al., 2015) and obesity prevention (Brust-Renck et al., 2017). While numerical health literacy and educational attainment typically correlate positively (Okan et al., 2019), this study's findings diverge from this trend, possibly due to a higher proportion of highly educated participants who may not represent interpretation difficulties at lower education levels. Nevertheless, previous research supports the findings, indicating that limited health literacy can negatively impact informed decision-making, regardless of educational attainment, as observed in colorectal cancer screening health communications (Kobayashi et al., 2014; Oldach & Katz, 2014; Smith et al., 2015; Smith et al., 2015). Despite over 80% of participants having a degree-level education in both phases, the leaflet's complexity appears to surpass their understanding. Acknowledging differences between phases A and B regarding interpretation difficulties and knowledge gaps within the leaflet is crucial. The variance may be explained by varying levels of attention given to the leaflet, with participants in phase B admitting to skipping uninteresting or irrelevant sections, potentially contributing to increased interpretation difficulties in phase A due to inattention. However, phase A's interaction with the leaflet may better represent actual leaflet recipients, aligning with previous research on incomplete engagement with leaflets in colorectal health communications (Ghanouni et al., 2017).

A noteworthy finding from the interviews was the significant impact of social influences, particularly mother figures, on women's interpretation of the leaflet, engagement, and future screening behaviour. This aligns with previous research highlighting the role of female family members in knowledge acquisition about the screening procedure (Sadler et al., 2013). The extent of this social influence is concerning, as it relies on parental prior experiences, which, if negative, may adversely affect future screening behaviour, as observed in studies of Japanese daughters approaching screening age (Egawa-Takata et al., 2016). This reliance on parental influence leaves young women vulnerable to misinformation about cervical screening, compromising their ability to make informed decisions (Southwell et al., 2019). Interventions aimed at educating mothers and encouraging them to recommend screening have shown to increase screening uptake (Egawa-Takata et al., 2018). Hence, future health communication materials should consider incorporating the influence of social factors, and further research could explore this aspect in intervention strategies.

The interview findings highlight the significant influence of health and cultural beliefs on leaflet interpretation, engagement, and motivation to engage. Aligning with previous research, young women perceived themselves to be at lower risk of cervical cancer (Sadler et al., 2013), with those from non-white ethnic backgrounds experiencing more interpretation difficulties (Okan et al., 2019). Cultural beliefs, such as abstinence before marriage, an acknowledged barriers to screening uptake (Marlow et al., 2015), seemingly hinder interpretation and engagement with the leaflet. Engagement with the HPV vaccine created a false sense of security among participants, with many incorrectly assuming that it protects against all types of HPV causing cervical cancer, consistent with previous research (Henderson et al., 2011; Sadler et al., 2013). However, this engagement did not significantly impact participants’ perception of cancer risk, contrasting with previous findings suggesting that vaccinated women are more likely to undergo screening (Kitchener et al., 2018). This may be attributed to differences in knowledge acquisition, as women with a better understanding of HPV and its association with cervical cancer risk are more likely to undergo screening (Chorley et al., 2017). The observed interpretation difficulties and knowledge gaps may contribute to the perception of higher protection and lower cancer risk.

The findings underscore that participants’ interpretation of cervical screening information is influenced by existing beliefs regarding low cancer risk and cultural beliefs, potentially linked to defensive information processing (van ‘t Riet & Ruiter, 2013). Some participants, holding beliefs of low cancer risk and high HPV vaccine protection, reported heightened convictions due to information in the leaflet, aligning with previous studies on health risk communications (Smith et al., 2016). A key issue highlighted by participants is the relevance of information, with beliefs about cancer risk, HPV vaccine protection, and cultural beliefs influencing disengagement with the cervical screening leaflet and service. Information relevance poses a challenge in ‘generalised’ health communications, such as leaflets (Bol et al., 2020, september; Kreuter et al., 1999; Noar et al., 2007). According to the elaboration likelihood model (Cacioppo & Petty, 1984), improving perceived personal relevance can foster user engagement, detailed processing, improved recall, and increased motivation for desired health behaviours (Bol et al., 2020, september). Tailored health information has proven effective in facilitating health behaviour change (Pourebrahim-Alamdari et al., 2021; Ryan et al., 2019, february). Tailoring cervical cancer information to address specific categories, such as low cancer risk perception and cultural beliefs, may enhance young women's engagement with screening health communications, as tailored information is perceived as personally relevant (Bol et al., 2020, september). It is noteworthy that previous attempts to increase cervical screening engagement through a tailored pre-notification leaflet (Kitchener et al., 2018) may have been constrained by a paper format, incongruent with the preferences for digital information among women under screening age. Tailoring information through digital means (e.g, tailored videos, information and access resources could be incorporated within the NHS app) may help increase uptake, supported by various previous studies (Huf, 2018; Huf et al., 2020).

Participants generally found the cervical screening leaflet impractical, inconvenient, and unappealing, contrary to previous research favouring paper leaflets as the preferred invitation method (Ryan et al., 2019). However, earlier studies did not explore participants’ post-engagement preferences, overlooking the potential for opinions to evolve. The study's findings on leaflet appeal aligned with prior research, emphasising that standard medical leaflets lack attractiveness to younger populations, who often prefer more abstract designs (Steele et al., 2011). Consistent with earlier research, the study identified clinical language and a lack of supportive or reassuring tones in the leaflet (Sadler et al., 2013). Therefore, a fundamental redesign of the leaflet is recommended to enhance engagement. Despite these drawbacks, participants positively received the diagrams in the leaflet, recognising their importance in enhancing understanding, consistent with previous research suggesting that visual health information is more effective in improving knowledge and attitudes towards screening. Thus, developing a visually appealing health communication tool may address the issue of young women's disengagement with the current leaflet.

Digital approaches have the potential to enhance young women's engagement with cervical screening health communication tools by offering convenience and presenting information in a format that aligns with their preferences. Previous research supports the preference and convenience of digital health communications (Ryan et al., 2019; Ulfa et al., 2019), and the effectiveness of audio/video techniques in improving knowledge acquisition and attitudes towards screening (Steele et al., 2011). Additionally, digital methods have shown potential to increase cervical screening uptake (Huf et al., 2020). However, a combination of health promotion methods, are likely to be more effective than a single approach. Incorporating multiple techniques with a digital foundation may ultimately enhance future engagement with the cervical screening programme (2019).

The study highlights a significant disparity between intention and behaviour regarding cervical screening, with young women acknowledging its importance yet delaying attendance. This aligns with previous research showing a 27% likelihood of screening uptake 18 months post-invitation (Kitchener et al., 2016, september 30; Kitchener et al., 2018). The findings underscore ambivalence towards screening importance and a deferred intention to engage, emphasising the need to bridge the intention-behaviour gap. Tailored interventions at the decision-making stage, upon receiving the invitation, are recommended to enhance engagement. Digital educational initiatives are suggested to address identified barriers, counter low cancer risk beliefs, and amplify the perceived importance of screening (Chorley et al., 2017; Vorsters et al., 2017). Additionally, implementing at-home screening test kits, supported by previous research (Campbell et al., 2020), shows promise in overcoming emotional barriers such as embarrassment and anxiety, thereby addressing critical factors hindering young women from attending screening appointments.

## Strengths and limitations

This study is the first to investigate how young women interpret the NHS cervical screening information leaflet and its impact on future screening behaviour. Using a mixed methods approach, researchers gained insight into participants’ perspectives on cervical screening materials and identified potential barriers or facilitators to decision-making. Focusing on young women nearing screening age minimises biases from past screening experiences. However, it's worth noting that participants may have approached the leaflet hypothetically, which could affect their interpretation compared to real-world decision-making (Von Wagner et al., 2010).

This study faces several limitations warranting consideration. Despite efforts to reach a wide audience through social media, the sample does not represent the diversity of the population Participant characteristics do not accurately represent the North East England population due to recruitment challenges amid the COVID-19 pandemic e.g. higher education level 87.5% in sample vs 14.5% in census data, and ethnicity whilst more evenly spread still not regionally representative of North East England (see supplementary file 1 Table 4 for full comparison between participant sample and census data for the North East) . Recruitment challenges during COVID also limited the number participants we were able to recruit during phase B. Despite pre-testing, the interpretation of specific item wording in phase A could impact participant performance, leading to potential misinterpretations. For instance, the absence of a specified time period for storing result records in the leaflet may have influenced participants to interpret an item as correct. The predictive validity of the multiple regression assessing motivation to engage was compromised by using a single-item measure as a dependent variable, despite its deemed suitability in previous research (Gardner et al., 2012; Presseau et al., 2011). Employing a multiple-item measure could have enhanced predictive validity (Diamantopoulos et al., 2012). As we were following Okan et al. (2019) strategy, data was not collected on what participants primary language was, which may have had as much impact upon interpretation of the leaflet as ethnicity. This warrants further investigation within future research. Furthermore, literacy was not measured specifically, numeracy was used as a partial measure of health literacy, future research should consider both measures for a true representation of health literacy’s impact. Despite the 4-week interval between study phases, prior interactions might have biased participants’ perceptions, potentially affecting the accuracy of initial interpretation and diminishing interpretation difficulties in phase B. Additionally, 16% of participants in phase A had previously received their leaflets which calls into question the validity of the findings as they may not reflect their initial interpretation when first engaging with the leaflet, this may also introduce potential bias of their interpretation due to the women potentially previously engaging with the content in the leaflet. Participants’ independent research on cervical screening through online sources or discussions with friends and family may have influenced their interpretation of the leaflet. The use of an unmarked think-aloud protocol, while attempts were made to minimise its impact, may have hindered the identification of misinterpretations, as marked protocols are considered more effective (Crain-Thoreson et al., 1997). Lastly, the TDF was not fully leveraged in designing phase B's interview schedule, potentially overlooking barriers and facilitators within participant characteristics across all TDF domains.

## Implications and future research

This study evaluates the NHS cervical screening leaflet, the main communication tool in the screening programme, noting strengths and weaknesses. Addressing these shortcomings could reduce information disparities among young women with lower health literacy and non-white ethnic backgrounds, promoting informed decision-making. While the focus is on the NHS England leaflet, the findings have wider relevance for improving other cervical screening communications, e.g. NHS website. Future policies should prioritise educational initiatives for young women and parents at the onset of HPV vaccine eligibility to counter perceptions of low cancer risk and enhance future screening uptake. Implementing a digital invitation featuring simplified gist representation, targeted behaviour change techniques (BCTs) (Michie et al., 2013), videos, and interactive tools can enhance education and promote screening behaviour. Despite cost-effectiveness of text messaging invites, incorporating successful behavioural messaging (Huf et al., 2020) and links to the NHS website or an app may prove more effective for engaging young women (Ryan et al., 2020), despite potential infrastructure costs (Huf et al., 2020).

Future research should have a two-fold focus: evaluating young women's understanding of cervical screening information from the NHS website and developing an educational intervention using a digital design that incorporates gist representation and BCTs to overcome barriers to screening uptake. These lines of enquiry will help inform decision-making on cervical screening participation and address inconsistencies in health communications.

## Conclusion

The study investigated young women's understanding, engagement, and impact of the current cervical screening leaflet. Results revealed persistent interpretation challenges, impracticality of the paper leaflet, and preference for digital health communications. Multiple barriers to comprehension, engagement, and future screening behaviour were identified. These findings offer insights to reduce disparities in informed decision-making and inform development of effective digital health communications to boost cervical screening uptake.

## Supplementary Material

Supplemental Material

Supplemental Material

Supplemental Material

Supplemental Material

## References

[CIT0001] 2011 Census Programme. (2009a). Final recommended questions for the 2011 census in England and Wales: Ethnic group. https://www.ons.gov.uk/census/2011census/howourcensusworks/howweplannedthe2011census/questionnairedevelopment/finalisingthe2011questionnaire

[CIT0002] 2011 Census Programme. (2009b). Final recommended questions for the 2011 Census in England and Wales: religion. https://www.ons.gov.uk/census/2011census/howourcensusworks/howweplannedthe2011census/questionnairedevelopment/finalisingthe2011questionnaire

[CIT0003] Ajzen I. (2006). Constructing a TPB questionnaire: Conceptual and methodological considerations. Retrieved from https://citeseerx.ist.psu.edu/document?repid=rep1&type=pdf&doi=0574b20bd58130dd5a961f1a2db10fd1fcbae95d

[CIT0004] Audina, W., Elfarianti, B., Daniati, L., Marpaung, M. R., Yulanda, E. S., Siahaan, E. Y., … Fasrini, U. U.. (2019). Health promotion intervention for increasing cervical cancer awareness screening: A systematic review. *Health Education*, *6*(12), 294–299.

[CIT0005] Bol, N., Smit, E. S., & Lustria, M. L. (2020, September). Tailored health communication: Opportunities and challenges in the digital era. *Digital health*, *6*, 1–3. 10.1177/2055207620958913 PMC7520919 33029355

[CIT0006] Braun, V., & Clarke, V. (2006, January 1). Using thematic analysis in psychology. *Qualitative Research in Psychology*, *3*(2), 77–101. 10.1191/1478088706qp063oa

[CIT0007] Brust-Renck, P. G., Reyna, V. F., Wilhelms, E. A., Wolfe, C. R., Widmer, C. L., Cedillos-Whynott, E. M., & Morant, A. K. (2017, August). Active engagement in a web-based tutorial to prevent obesity grounded in Fuzzy-Trace Theory predicts higher knowledge and gist comprehension. *Behavior Research Methods*, *49*(4), 1386–1398. 10.3758/s13428-016-0794-1 27531360 PMC5313384

[CIT0008] Cacioppo, J. T., & Petty, R. E. (1984, January). The elaboration likelihood model of persuasion. *Advances in consumer research*, *11*(1), 673–675.

[CIT0009] Campbell, H. E., Gray, A. M., Watson, J., Jackson, C., Moseley, C., Cruickshank, M. E., Kitchener, H. C., & Rivero-Arias, O. (2020, February). Preferences for interventions designed to increase cervical screening uptake in non-attending young women: How findings from a discrete choice experiment compare with observed behaviours in a trial. *Health Expectations*, *23*(1), 202–211. 10.1111/hex.12992 31659850 PMC6978852

[CIT0010] Cancer Research UK. (2020). Cervical cancer progress falters as screening uptake hits record lows. https://www.cancerresearchuk.org/about-us/cancer-news/press-release/2020-01-22-cervical-cancer-progress-falters-as-screening-uptake-hits-record-lows

[CIT0011] Cane, J., O’Connor, D., & Michie, S. (2012, December). Validation of the theoretical domains framework for use in behaviour change and implementation research. *Implementation Science*, *7*(1), 1–7. 10.1186/1748-5908-7-37 PMC3483008 22530986

[CIT0012] Chorley, A. J., Hirst, Y., Vrinten, C., von Wagner, C., Wardle, J., & Waller, J. (2018, June). Public understanding of the purpose of cancer screening: A population-based survey. *Journal of Medical Screening*, *25*(2), 64–69. 10.1177/0969141317699440 28530514 PMC5956561

[CIT0013] Chorley, A. J., Marlow, L. A., Forster, A. S., Haddrell, J. B., & Waller, J. (2017, February). Experiences of cervical screening and barriers to participation in the context of an organised programme: A systematic review and thematic synthesis. *Psycho-oncology*, *26*(2), 161–172. 10.1002/pon.4126 27072589 PMC5324630

[CIT0014] Couch, B. A., Hubbard, J. K., & Brassil, C. E. (2018, June 1). Multiple–true–false questions reveal the limits of the multiple–choice format for detecting students with incomplete understandings. *BioScience*, *68*(6), 455–463. 10.1093/biosci/biy037

[CIT0015] Crain-Thoreson, C., Lippman, M. Z., & McClendon-Magnuson, D. (1997, December). Windows on comprehension: Reading comprehension processes as revealed by two think-aloud procedures. *Journal of Educational Psychology*, *89*(4), 579. 10.1037/0022-0663.89.4.579

[CIT0016] de Bruin, W. B., Downs, J. S., Fischhoff, B., & Palmgren, C. (2007, July 20). Development and evaluation of an HIV/AIDS knowledge measure for adolescents focusing on misconceptions. *Journal of HIV/AIDS Prevention in Children & Youth*, *8*(1), 35–57. 10.1300/J499v08n01_03

[CIT0017] de Bruin, W. B., & Fischhoff, B. (2000, June 1). The effect of question format on measured HIV/AIDS knowledge: Detention center teens, high school students, and adults. *AIDS Education and Prevention*, *12*(3), 187. 10926123

[CIT0018] Diamantopoulos, A., Sarstedt, M., Fuchs, C., Wilczynski, P., & Kaiser, S. (2012, May). Guidelines for choosing between multi-item and single-item scales for construct measurement: A predictive validity perspective. *Journal of the Academy of Marketing Science*, *40*(3), 434–449. 10.1007/s11747-011-0300-3

[CIT0019] Douglas, E., Waller, J., Duffy, S. W., & Wardle, J. (2016, June). Socioeconomic inequalities in breast and cervical screening coverage in England: Are we closing the gap? *Journal of Medical Screening*, *23*(2), 98–103. 10.1177/0969141315600192 26377810 PMC4855247

[CIT0020] Egawa-Takata, T., Ueda, Y., Morimoto, A., Tanaka, Y., Yagi, A., Terai, Y., Ohmichi, M., Ichimura, T., Sumi, T., Murata, H., & Okada, H. (2018, March 5). Motivating mothers to recommend their 20-year-old daughters receive cervical cancer screening: A randomized study. *Journal of Epidemiology*, *28*(3), 156–160. 10.2188/jea.JE20160155 29129894 PMC5821693

[CIT0021] Egawa-Takata, T., Ueda, Y., Tanaka, Y., Morimoto, A., Kubota, S., Yagi, A., Terai, Y., Ohmichi, M., Ichimura, T., Sumi, T., & Murata, H. (2016, October). Mothers’ attitudes in Japan regarding cervical cancer screening correlates with intention to recommend cervical cancer screening for daughters. *International Journal of Clinical Oncology*, *21*(5), 962–968. 10.1007/s10147-016-0970-4 26968588

[CIT0022] Ericsson, K. A., & Simon, H. A.. (1984). *Protocol analysis: Verbal reports as data*. Cambridge, MA: Bradford books/MIT Press.

[CIT0023] Falcaro, M., Castañon, A., Ndlela, B., Checchi, M., Soldan, K., Lopez-Bernal, J., Elliss-Brookes, L., & Sasieni, P. (2021, December 4). The effects of the national HPV vaccination programme in England, UK, on cervical cancer and grade 3 cervical intraepithelial neoplasia incidence: A register-based observational study. *The Lancet*, *398*(10316), 2084–2092. 10.1016/S0140-6736(21)02178-4 34741816

[CIT0024] Francis, J. J., Johnston, M., Robertson, C., Glidewell, L., Entwistle, V., Eccles, M. P., & Grimshaw, J. M. (2010, December 1). What is an adequate sample size? *Operationalising data saturation for theory-based interview studies. Psychology and health*, *25*(10), 1229–1245. 20204937 10.1080/08870440903194015

[CIT0025] Garcia-Retamero, R., & Cokely, E. T. (2017, June). Designing visual aids that promote risk literacy: A systematic review of health research and evidence-based design heuristics. *Human Factors*, *59*(4), 582–627. 10.1177/0018720817690634 28192674

[CIT0026] Gardner, B., de Bruijn, G. J., & Lally, P. (2012, September). Habit, identity, and repetitive action: A prospective study of binge-drinking in UK students. *British Journal of Health Psychology*, *17*(3), 565–581. 10.1111/j.2044-8287.2011.02056.x 22107605

[CIT0027] Ghanouni, A., Renzi, C., & Waller, J. (2017, December). A cross-sectional survey assessing factors associated with Reading cancer screening information: Previous screening behaviour, demographics and decision-making style. *BMC Public Health*, *17*(1), 1–2. 10.1186/s12889-017-4224-9 28420378 PMC5395826

[CIT0028] Gigerenzer, G., & Edwards, A. (2003, September 25). Simple tools for understanding risks: From innumeracy to insight. *BMJ*, *327* (7417):741–744. 14512488 10.1136/bmj.327.7417.741 PMC200816

[CIT0029] Gurmankin, A. D., Baron, J., & Armstrong, K. (2004, June). The effect of numerical statements of risk on trust and comfort with hypothetical physician risk communication. *Medical Decision Making*, *24*(3), 265–271. 10.1177/0272989X04265482 15155015

[CIT0030] Henderson, L., Clements, A., Damery, S., Wilkinson, C., Austoker, J., & Group, S. W. (2011, March). A false sense of security’? Understanding the role of the HPV vaccine on future cervical screening behaviour: A qualitative study of UK parents and girls of vaccination age. *Journal of Medical Screening*, *18*(1), 41–45. 10.1258/jms.2011.010148 21536816 PMC3104818

[CIT0031] Huf, S. (2018). The use of behavioural sciences in targeted health messages to improve the participation in cervical and breast screening programmes. Diss. Imperial College London.

[CIT0032] Huf, S., Kerrison, R. S., King, D., Chadborn, T., Richmond, A., Cunningham, D., Friedman, E., Shukla, H., Tseng, F. M., Judah, G., & Darzi, A. (2020, October 1). Behavioral economics informed message content in text message reminders to improve cervical screening participation: Two pragmatic randomized controlled trials. *Preventive Medicine*, *139*, 106170. 32610059 10.1016/j.ypmed.2020.106170

[CIT0033] Jenkins, S. C., Harris, A. J., & Lark, R. M. (2019, May 4). When unlikely outcomes occur: The role of communication format in maintaining communicator credibility. *Journal of Risk Research*, *22*(5), 537–554. 10.1080/13669877.2018.1440415

[CIT0034] Johnstone, C. J., Bottsford-Miller, N. A., & Thompson, S. J. (2006, August). Using the think aloud method (cognitive labs) to evaluate test design for students with disabilities and English language learners. Technical Report 44. National Center on Educational Outcomes, University of Minnesota.

[CIT0035] Jo’s Cervical Cancer Trust. (2017). Embarrassment preventing young women from attending a test that could save their life*.* https://www.jostrust.org.uk/node/573478

[CIT0036] Jo’s Cervical Cancer Trust. (2018). Body shame responsible for young women not attending smear tests. https://www.jostrust.org.uk/node/1073042

[CIT0037] Jo's Cervical Cancer Trust. (2019). Smear test fears causing hidden health crisis for Millennials. https://www.jostrust.org.uk/node/1076499

[CIT0038] Kelly, S., Martin, S., Kuhn, I., Cowan, A., Brayne, C., & Lafortune, L. (2016, January 27). Barriers and facilitators to the uptake and maintenance of healthy behaviours by people at mid-life: A rapid systematic review. *PLoS One*, *11*(1), e0145074. 26815199 10.1371/journal.pone.0145074 PMC4731386

[CIT0039] Kitchener, H., Gittins, M., Cruickshank, M., Moseley, C., Fletcher, S., Albrow, R., Gray, A., Brabin, L., Torgerson, D., Crosbie, E. J., & Sargent, A. (2018, June). A cluster randomized trial of strategies to increase uptake amongst young women invited for their first cervical screen: The STRATEGIC trial. *Journal of Medical Screening*, *25*(2), 88–98. 10.1177/0969141317696518 28530513 PMC5956569

[CIT0040] Kitchener, H. C., Gittins, M., Rivero-Arias, O., Tsiachristas, A., Cruickshank, M., Gray, A., … Roberts, C. (2016, September 30). A cluster randomised trial of strategies to increase cervical screening uptake at first invitation (STRATEGIC). *Health Technology Assessment*, *20*(68), 1–138. 10.3310/hta20680 PMC5046076 27632816

[CIT0041] Kobayashi, L. C., Wardle, J., & von Wagner, C. (2014, April 1). Limited health literacy is a barrier to colorectal cancer screening in England: Evidence from the English longitudinal study of ageing. *Preventive Medicine*, *61*, 100–105. 10.1016/j.ypmed.2013.11.012 24287122 PMC3969575

[CIT0042] Kreuter, M. W., Strecher, V. J., & Glassman, B. (1999, December). One size does not fit all: The case for tailoring print materials. *Annals of Behavioral Medicine*, *21*(4), 276–283. 10.1007/BF02895958 10721433

[CIT0043] Landy, R., Pesola, F., Castañón, A., & Sasieni, P. (2016, October). Impact of cervical screening on cervical cancer mortality: Estimation using stage-specific results from a nested case–control study. *British Journal of Cancer*, *115*(9), 1140–1146. 10.1038/bjc.2016.290 27632376 PMC5117785

[CIT0044] Landy, R., Sasieni, P. D., Mathews, C., Wiggins, C. L., Robertson, M., McDonald, Y. J., Goldberg, D. W., Scarinci, I. C., Cuzick, J., & Wheeler, C. M. (2020, August 1). New Mexico HPV Pap Registry Steering Committee. Impact of screening on cervical cancer incidence: A population-based case–control study in the United States. *International Journal of Cancer*, *147*(3), 887–896. 10.1002/ijc.32826 31837006 PMC7282928

[CIT0045] Lucero, K. S., & Chen, P. (2020, January 1). What do reinforcement and confidence have to do with it? *A systematic pathway analysis of knowledge, competence, confidence, and intention to change. Journal of European CME*, *9*(1), 1834759. 33133769 10.1080/21614083.2020.1834759 PMC7580825

[CIT0046] Marlow, L., Forster, A. S., McBride, E., Rockliffe, L., Kitchener, H., & Waller, J. (2020, December 1). Information needs among women taking part in primary HPV screening in England: A content analysis. *BMJ Open*, *10*(12), e044630. 10.1136/bmjopen-2020-044630 PMC7745520 33323451

[CIT0047] Marlow, L. A., Waller, J., & Wardle, J. (2015, October 1). Barriers to cervical cancer screening among ethnic minority women: A qualitative study. *Journal of Family Planning and Reproductive Health Care*, *41*(4), 248–254. 10.1136/jfprhc-2014-101082 25583124 PMC4621371

[CIT0048] Mesher, D., Cuschieri, K., Hibbitts, S., Jamison, J., Sargent, A., Pollock, K. G., Powell, N., Wilson, R., McCall, F., Fiander, A., & Soldan, K. (2015, February 1). Type-specific HPV prevalence in invasive cervical cancer in the UK prior to national HPV immunisation programme: Baseline for monitoring the effects of immunisation. *Journal of Clinical Pathology*, *68*(2), 135–140. 10.1136/jclinpath-2014-202681 25410654

[CIT0049] Michie, S., Richardson, M., Johnston, M., Abraham, C., Francis, J., Hardeman, W., Eccles, M. P., Cane, J., & Wood, C. E. (2013, August 1). The behavior change technique taxonomy (v1) of 93 hierarchically clustered techniques: Building an international consensus for the reporting of behavior change interventions. *Annals of Behavioral Medicine*, *46*(1), 81–95. 10.1007/s12160-013-9486-6 23512568

[CIT0050] National Readership Survey. (2021). Social grade. http://www.nrs.co.uk/nrs-print/lifestyle-and-classification-data/social-grade/

[CIT0051] NHS. (2019). Cervical Screening Programme, England - 2018-19. https://files.digital.nhs.uk/56/6FF6AB/nhs-cerv-scre-prog-eng-2018-19-report.pdf

[CIT0052] NHS. (2021). Cervical screening*.* https://www.nhs.uk/conditions/cervical-screening/

[CIT0053] Noar, S. M., Benac, C. N., & Harris, M. S. (2007). Does tailoring matter? Meta-analytic review of tailored print health behavior change interventions. *Psychological Bulletin*, *133*(4), 673. 10.1037/0033-2909.133.4.673 17592961

[CIT0054] Office for Health Improvement and Disparities. (2023). Public health profiles. https://fingertips.phe.org.uk

[CIT0055] Okan, Y., Petrova, D., Smith, S. G., Lesic, V., & de Bruin W, B. (2019, October). How do women interpret the NHS information leaflet about cervical cancer screening? *Medical Decision Making*, *39*(7), 738–754. 10.1177/0272989X19873647 31556840 PMC6843617

[CIT0056] Oldach, B. R., & Katz, M. L. (2014, February 1). Health literacy and cancer screening: A systematic review. *Patient Education and Counseling*, *94*(2), 149–157. 10.1016/j.pec.2013.10.001 24207115 PMC3946869

[CIT0057] Olsson, H. (2014, August 1). Measuring overconfidence: Methodological problems and statistical artifacts. *Journal of Business Research*, *67*(8), 1766–1770. 10.1016/j.jbusres.2014.03.002

[CIT0058] Patel, H., Moss, E. L., & Sherman, S. M. (2018, June). HPV primary cervical screening in England: Women's awareness and attitudes. *Psycho-Oncology*, *27*(6), 1559–1564. 10.1002/pon.4694 29521462

[CIT0059] Petrova, D., Kostopoulou, O., Delaney, B. C., Cokely, E. T., & Garcia-Retamero, R. (2018, April). Strengths and gaps in physicians’ risk communication: A scenario study of the influence of numeracy on cancer screening communication. *Medical Decision Making*, *38*(3), 355–365. 10.1177/0272989X17729359 28884617

[CIT0060] Pourebrahim-Alamdari, P., Mehrabi, E., Nikkhesal, N., Nourizadeh, R., Esmaeilpour, K., & Mousavi, S. (2021). Effectiveness of motivationally tailored interventions on cervical cancer screening: A systematic review and meta-analysis. *Int J Womens Health Reprod Sci*, *9*(2), 86–90. 10.15296/ijwhr.2021.16

[CIT0061] Presseau, J., Francis, J. J., Campbell, N. C., & Sniehotta, F. F. (2011, December). Goal conflict, goal facilitation, and health professionals’ provision of physical activity advice in primary care: An exploratory prospective study. *Implementation Science*, *6*(1), 1–9. 10.1186/1748-5908-6-73 21762486 PMC3224555

[CIT0062] Ravindran, R., Cotton, S., & Cruickshank, M. (2020, January). Women's preferences for communication with the cervical screening programme: A qualitative study. *Cytopathology*, *31*(1), 47–52. 10.1111/cyt.12783 31677212

[CIT0063] Reyna, V. F. (2008, November). A theory of medical decision making and health: Fuzzy trace theory. *Medical Decision Making*, *28*(6), 850–865. 10.1177/0272989X08327066 19015287 PMC2617718

[CIT0064] Ryan, K., Dockray, S., & Linehan, C. (2019, February). A systematic review of tailored eHealth interventions for weight loss. *Digital Health*, *5*, 1–23 10.1177/2055207619826685. PMC6366004 30783535

[CIT0065] Ryan, M., Marlow, L., Forster, A., Ruwende, J., & Waller, J. (2020, June). Offering an app to book cervical screening appointments: A service evaluation. *Journal of Medical Screening*, *27*(2), 85–89. 10.1177/0969141319871312 31500520 PMC7222961

[CIT0066] Ryan, M., Waller, J., & Marlow, L. A. (2019, July 1). Could changing invitation and booking processes help women translate their cervical screening intentions into action? A population-based survey of women’s preferences in Great Britain. *BMJ Open*, *9*(7), e028134. 10.1136/bmjopen-2018-028134 PMC6629419 31300499

[CIT0067] Sadler, L., Albrow, R., Shelton, R., Kitchener, H., & Brabin, L. (2013). Development of a pre-notification leaflet to encourage uptake of cervical screening at first invitation: A qualitative study. *Health Education Research*, *28*(5), 793–802. 10.1093/her/cys103 23111151

[CIT0068] Salter, J. (2014). *Behind the screen: Revealing the true cost of cervical cancer*. Demos.

[CIT0069] Schiffman, M., Kinney, W. K., Cheung, L. C., Gage, J. C., Fetterman, B., Poitras, N. E., Lorey, T. S., Wentzensen, N., Befano, B., Schussler, J., & Katki, H. A. (2018, May 1). Relative performance of HPV and cytology components of cotesting in cervical screening. *JNCI: Journal of the National Cancer Institute*, *110*(5), 501–508. 10.1093/jnci/djx225 29145648 PMC6279277

[CIT0070] Schwartz, L. M., Woloshin, S., Black, W. C., & Welch, H. G. (1997, December 1). The role of numeracy in understanding the benefit of screening mammography. *Annals of Internal Medicine*, *127*(11), 966–972. 10.7326/0003-4819-127-11-199712010-00003 9412301

[CIT0071] Smith, S. G., Kobayashi, L. C., Wolf, M. S., Raine, R., Wardle, J., & Von Wagner, C. (2016, August). The associations between objective numeracy and colorectal cancer screening knowledge, attitudes and defensive processing in a deprived community sample. *Journal of Health Psychology*, *21*(8), 1665–1675. 10.1177/1359105314560919 25512199

[CIT0072] Smith, S. G., Raine, R., Obichere, A., Wolf, M. S., Wardle, J., & von Wagner, C. (2015, April). The effect of a supplementary (‘gist-based’) information leaflet on colorectal cancer knowledge and screening intention: A randomized controlled trial. *Journal of Behavioral Medicine*, *38*(2), 261–272. 10.1007/s10865-014-9596-z 25253443 PMC4353886

[CIT0073] Smith, S. G., Vart, G., Wolf, M. S., Obichere, A., Baker, H. J., Raine, R., Wardle, J., & von Wagner, C. (2015, October). How do people interpret information about colorectal cancer screening: Observations from a think-aloud study. *Health Expectations*, *18*(5), 703–714. 10.1111/hex.12117 23910930 PMC5060830

[CIT0074] Southwell, B. G., Niederdeppe, J., Cappella, J. N., Gaysynsky, A., Kelley, D. E., Oh, A., Peterson, E. B., & Chou, W. Y. (2019, August 1). Misinformation as a misunderstood challenge to public health. *American Journal of Preventive Medicine*, *57*(2), 282–285. 10.1016/j.amepre.2019.03.009 31248741

[CIT0075] Steele, M., Dow, L., & Baxter, G. (2011, December 1). Promoting public awareness of the links between lifestyle and cancer: A controlled study of the usability of health information leaflets. *International Journal of Medical Informatics*, *80*(12), e214–e229. 10.1016/j.ijmedinf.2011.08.012 22020199

[CIT0076] Ulfa, M., Tahir, A. M., & Ulfa, M. (2019). The effects of audio visual information and leaflets towards increasing knowledge, mother’s demeanour on cervical cancer and visual inspection With acetic acid (via) in Sudiang Health Center, Makassar. *East African Scholars Journal of Medical Sciences*, *4421*(6), 319–325.

[CIT0077] van der Meij, A. E., Damman, O. C., Uiters, E., & Timmermans, D. R. (2019, July 1). What benefits and harms are important for a decision about cervical screening? A study of the perspective of different subgroups of women. *Patient Preference and Adherence*, *13*, 1005–1017. 10.2147/PPA.S193522. 31303748 PMC6611716

[CIT0078] van ‘t Riet, J., & Ruiter, R. A. (2013, May 1). Defensive reactions to health-promoting information: An overview and implications for future research. *Health Psychology Review*, *7*(sup1), S104–S136. 10.1080/17437199.2011.606782

[CIT0079] Verdoodt, F., Jentschke, M., Hillemanns, P., Racey, C. S., Snijders, P. J., & Arbyn, M. (2015, November 1). Reaching women who do not participate in the regular cervical cancer screening programme by offering self-sampling kits: A systematic review and meta-analysis of randomised trials. *European Journal of Cancer*, *51*(16), 2375–2385. 10.1016/j.ejca.2015.07.006 26296294

[CIT0080] Visschers, V. H., Meertens, R. M., Passchier, W. W., & De Vries, N. N. (2009, February). Probability information in risk communication: A review of the research literature. *Risk Analysis: An International Journal*, *29*(2), 267–287. 10.1111/j.1539-6924.2008.01137.x 19000070

[CIT0081] Von Wagner, C., Semmler, C., Power, E., & Good, A. (2010, March). What matters when deciding whether to participate in colorectal cancer screening? The moderating role of time perspective. *Journal of Applied Biobehavioral Research*, *15*(1), 20–30. 10.1111/j.1751-9861.2010.00050.x

[CIT0082] Vorsters, A., Arbyn, M., Baay, M., Bosch, X., de Sanjosé, S., Hanley, S., Karafillakis, E., Lopalco, P. L., Pollock, K. G., Yarwood, J., & Van Damme, P. (2017, December 1). Overcoming barriers in HPV vaccination and screening programs. *Papillomavirus Research*, *4*, 45–53. 10.1016/j.pvr.2017.07.001 29179869 PMC7268103

[CIT0083] Wittink, H., & Oosterhaven, J. (2018, December 1). Patient education and health literacy. *Musculoskeletal Science and Practice*, *38*, 120–127. 10.1016/j.msksp.2018.06.004 30017902

