# Supplementary material for: How do young women approaching screening age interpret the NHS cervical screening leaflet? A mixed methods study of identifying interpretation difficulties, barriers, facilitators, and leaflet interpretation, engagement and future screening behaviour
Source: Health Psychol Behav Med. 2024 May 30;12(1):2361005. doi: 10.1080/21642850.2024.2361005 (PMC11146246; doi:10.1080/21642850.2024.2361005)
Supplement: Supplemental Material [file RHPB_A_2361005_SM6192.docx]

Supplementary file 4: Phase B Analysis Strategy and Theme Development

The analysis strategy of the transcripts was conducted within 2 phases; firstly, for the cognitive think-aloud and leaflet utility sections the coding strategy utilised Braun and Clarke [1] thematic analysis 6-stage approach by first (1) data familiarisation, followed by (2) generating codes which were close with the dataset (3) theme searching, (4) theme reviewing, (5) naming and identifying themes and finally (6) producing a written report of the analysis. For items eliciting responses on facilitators or barriers of leaflet interpretation, engagement, or future screening behaviour, the coding strategy utilised thematic analysis but was informed by the Theory Domain Framework (TDF) [2], with developed themes being refined and mapped within the relevant domains within the TDF. At this stage, any initial codes within the cognitive think-aloud or leaflet utility section which referenced barriers or facilitators of leaflet interpretation, engagement or future screening behaviour were also mapped and refined using the TDF. This strategy was employed in order to allow for the identification of barriers or facilitators within the dataset which were not related to participant characteristics and allow for themes within the dataset which may not be covered by the TDF, therefore providing an in-depth explanation about the participants experiences of leaflet interpretation, engagement and future potential screening behaviour.

To ensure that credibility, validity, and trustworthiness was maintained throughout the coding, theme identification, and interpretation of the data, the following steps were taken. Transcripts were reviewed by the participants to ascertain the accuracy in capturing their responses, and whether the resultant themes adequately reflected the behaviour being assessed in the current study. Rich descriptions of participant accounts are provided within this paper to support the findings of this current study. Also, a thorough audit trail has been documented by the researcher to provide evidence of the decisions made throughout the study, including coding and theme development to ensure that the researchers interpretation was not subject to preconceived biases [3]. Finally, between methods triangulation has been utilised within the current study, with the triangulation point occurring within the discussion section of this report after separate analysis. By using both quantitative and qualitative analysis, increased scope, depth and consistency of the methodological proceedings, therefore increase the credibility and trustworthiness of the current study’s findings [4, 5].

1. Braun V, Clarke V. Using thematic analysis in psychology. Qualitative research in psychology. 2006 Jan 1;3(2):77-101.
2. Cane J, O’Connor D, Michie S. Validation of the theoretical domains framework for use in behaviour change and implementation research. Implementation science. 2012 Dec;7:1-7
3. Noble, H., & Smith, J. (2015). Issues of validity and reliability in qualitative research. *Evidence-based nursing*, *18*(2), 34-35.
4. Guion, L. A., Diehl, D. C., & McDonald, D. (2011). Triangulation: Establishing the validity of qualitative studies. *EDIS, 2011*(8), 3-3.
5. Flick, U. (2018). Triangulation in data collection. *The SAGE handbook of qualitative data collection*, 527-544.
