# Supplementary material for: How do young women approaching screening age interpret the NHS cervical screening leaflet? A mixed methods study of identifying interpretation difficulties, barriers, facilitators, and leaflet interpretation, engagement and future screening behaviour
Source: Health Psychol Behav Med. 2024 May 30;12(1):2361005. doi: 10.1080/21642850.2024.2361005 (PMC11146246; doi:10.1080/21642850.2024.2361005)
Supplement: Supplemental Material [file RHPB_A_2361005_SM6191.docx]

Supplementary file 3: Phase A data coding and analysis strategy

Confidence of accuracy variable was scored by calculating participants mean confidence score across all 19 interpretation items, with any missing confidence ratings not included within mean confidence calculation (maximum of 1 missing confidence rating). The motivation to engage variable was reverse scored due to response layout within the Qualtrics survey (not important (1) to very important (5)).Categorical predictor variables of education (higher vs other), ethnicity (white vs other), religion (religious vs non-religious), first language (English vs other) and HPV vaccine engagement (yes vs no – unsure coded as no) were split into 2 levels coded as 0 and 1 respectively. Participant social grade was calculated by the NRS classification system [1] using chief income occupation status to sort into relevant categories (A, B, C1, C2, D and E – see table 5 for category definitions), before being split into 2 levels of higher (A, B and C1) vs lower (C2, D and E) with same coding strategy employed as previous categorical variables. Numeracy was entered as a continuous variable (scores of 0-3, addition of correct responses), and cancer risk perception entered as an ordinal variable (1 low risk – 5 high risk).

Despite motivation to engage being an ordinal single measure variable, multiple regression was deemed an appropriate analysis strategy as an ordinal dependent variable with 5 or more categories can be used as a continuous variable within regression analyses [2, 3], and can be a single use measure and not impact upon the feasibility to conduct multiple linear regressions successfully [4, 5]. Further analysis of individual interpretation and confidence items were conducted to determine whether specific areas of the leaflet are causing interpretation problems, this was done by calculating the percentage of correct responses to individual interpretation items and mean confidence level for that item (see table 3). Correlational analyses were conducted to examine the relationship between interpretation accuracy, confidence of accuracy and motivation to engage. Again, due to the motivation to engage DV being ordinal, spearman’s rho correlational analysis was deemed as the most appropriate to conduct. Finally, A chi square analysis was conducted to examine the association between cancer risk perception and HPV vaccine engagement due to parametric analyses i.e., correlations not being suitable for categorical data (see supplementary tables 5 & 6).

References

1. National Readership Survey. Social Grade*.* 2021. Available from <http://www.nrs.co.uk/nrs-print/lifestyle-and-classification-data/social-grade/>
2. Norman G. Likert scales, levels of measurement and the “laws” of statistics. Advances in health sciences education. 2010 Dec;15:625-32.
3. Sullivan GM, Artino Jr AR. Analyzing and interpreting data from Likert-type scales. Journal of graduate medical education. 2013 Dec 1;5(4):541-2.
4. Presseau J, Francis JJ, Campbell NC, Sniehotta FF. Goal conflict, goal facilitation, and health professionals' provision of physical activity advice in primary care: An exploratory prospective study. Implementation Science. 2011 Dec;6:1-9.
5. Gardner B, de Bruijn GJ, Lally P. Habit, identity, and repetitive action: A prospective study of binge‐drinking in UK students. British journal of health psychology. 2012 Sep;17(3):565-81.
