# Supplementary material for: How do young women approaching screening age interpret the NHS cervical screening leaflet? A mixed methods study of identifying interpretation difficulties, barriers, facilitators, and leaflet interpretation, engagement and future screening behaviour
Source: Health Psychol Behav Med. 2024 May 30;12(1):2361005. doi: 10.1080/21642850.2024.2361005 (PMC11146246; doi:10.1080/21642850.2024.2361005)
Supplement: Supplemental Material [file RHPB_A_2361005_SM6190.docx]

Supplementary file 2: Cognitive think-aloud protocol and task participant instructions

*Cognitive think-aloud protocol*

Instructions for the cognitive think-aloud task were adapted from Crain-Thoreson and colleagues [1] and Okan and colleagues [2]. A leaflet about an unrelated topic was also incorporated (Leaflet can be retrieved from https://www.healthcheck.nhs.uk/commissioners-and-providers/marketing/leaflets/) due to following recommended procedures of requiring participants to practice and successfully completing 3 utterances before the interview began [3]. The unmarked protocol consisted of participants reading through the leaflet one section at a time. If participants were silent, or did not make an utterance after 3 seconds, they were prompted via the researcher reminding the participant to ‘keep talking’. The researcher decided upon this neutral que to refrain from potentially biasing the participant by introducing external ideas to the internal cognitive process [4].

*Cognitive think-aloud participant instructions*

During the interview, there will be a section where you will be asked to read through the cervical screening leaflet section by section. As the research is interested in that you are thinking whilst reading through the leaflet, I will therefore ask you to think aloud whilst you are reading.

To think aloud means to tell the researcher everything you are thinking as you are reading the information, it doesn’t matter what it is, there are no right and wrong answers in this section. An example may be wanting to say what you think the text is saying or make a comment on something which is perhaps confusing or unfamiliar. You could also perhaps make suggestions if you had any ideas of what could make the information clearer. Overall, you just want to act as if you are in a room alone speaking to yourself. I will not speak unless you continue to read without verbalising your thoughts for a short period of time, in such case I will remind you to keep talking. However, if there is anything you are uncomfortable with, or don’t wish to talk about within any section of the leaflet it can be skipped, and we can move on to the next section.

You will be given time to practice with a different leaflet before we start the interview and to ask any questions.

References

1. Crain-Thoreson C, Lippman MZ, McClendon-Magnuson D. Windows on comprehension: Reading comprehension processes as revealed by two think-aloud procedures. Journal of Educational Psychology. 1997 Dec;89(4):579.
2. Okan Y, Petrova D, Smith SG, Lesic V, Bruine de Bruin W. How do women interpret the NHS information leaflet about cervical cancer screening?. Medical Decision Making. 2019 Oct;39(7):738-54.
3. Fox MC, Ericsson KA, Best R. Do procedures for verbal reporting of thinking have to be reactive? A meta-analysis and recommendations for best reporting methods. Psychological bulletin. 2011 Mar;137(2):316.
4. Johnstone CJ, Bottsford-Miller NA, Thompson SJ. Using the Think Aloud Method (Cognitive Labs) to Evaluate Test Design for Students with Disabilities and English Language Learners. Technical Report 44. National Center on Educational Outcomes, University of Minnesota. 2006 Aug.
