# Supplementary material for: How do young women approaching screening age interpret the NHS cervical screening leaflet? A mixed methods study of identifying interpretation difficulties, barriers, facilitators, and leaflet interpretation, engagement and future screening behaviour
Source: Health Psychol Behav Med. 2024 May 30;12(1):2361005. doi: 10.1080/21642850.2024.2361005 (PMC11146246; doi:10.1080/21642850.2024.2361005)
Supplement: Supplemental Material [file RHPB_A_2361005_SM6185.docx]

Supplementary file 1: Tables

**Table 1**: Phase A survey items

| Item | Response |
| --- | --- |
| What is your age? | Open response |
| What is your education level? | No formal qualifications/GCSE or equivalent/A level of equivalent/Higher education or equivalent |
| What is your ethic group? | White/Mixed-multiple ethnic groups/Asian or Asian British/Black, African, Caribbean, Black British/Other Ethnic group – please specify (open response) |
| What is your religion? | No religion/Christian (all denominations)/ Buddhist/Hindu/Jewish/Muslim/Sikh/any other religion – please specify (open response) |
| Do you currently practice your religion? | Yes/No |
| Which of the following best describes your employment status? | Semi or unskilled manual worker (e.g., caretaker, park keeper, shop assistant etc.)/Skilled manual worker (e.g., bricklayer, carpenter, plumber, painter, pub or bar worker etc)/Supervisory or clerical, junior managerial, professional, Administrative (e.g., office worker, student doctor, foreman with 25+ employees, sales persons, etc)/Intermediate managerial, Professional, Administrative (e.g., newly qualified (under 3 years) doctor, board director of small organisation, middle manager in a large organisation, local government etc)/Higher managerial, Professional, Administrative (e.g., doctor, solicitor, board director in a large organisation 200+ employees, top level civil servant etc)/Student/ Casual worker (not in permanent employment/Housewife (homemaker)/Retired and living on state pension/Retired and not living on state pension/Unemployed or not working due to long term sickness/Full-time carer of other household member |
| Are you the chief income earner in your household? | Yes/No |
| Which one of the following categories best describes the employment status of the chief income earner in your household? | Semi or unskilled manual worker (e.g., caretaker, park keeper, shop assistant etc.)/Skilled manual worker (e.g., bricklayer, carpenter, plumber, painter, pub or bar worker etc)/Supervisory or clerical, junior managerial, professional, Administrative (e.g., office worker, student doctor, foreman with 25+ employees, sales persons, etc)/Intermediate managerial, Professional, Administrative (e.g., newly qualified (under 3 years) doctor, board director of small organisation, middle manager in a large organisation, local government etc)/Higher managerial, Professional, Administrative (e.g., doctor, solicitor, board director in a large organisation 200+ employees, top level civil servant etc)/Student/ Casual worker (not in permanent employment/Housewife (homemaker)/Retired and living on state pension/Retired and not living on state pension/Unemployed or not working due to long term sickness/Full-time carer of other household member |
| Is English your first language? | Yes/No |
| Have you received the HPV vaccination? | Yes/No/Unsure |
| Did you refuse the HPV vaccine when offered? Or was there another reason for not receiving the vaccine? | Open response |
| How did you receive the HPV vaccine? (e.g., school, Doctor’s surgery etc) | Open response |
| Have you received an invitation to the cervical screening programme with the information pack? | Yes/No |
| Do you know anyone who has ever been diagnosed with cervical cancer? | Yes/No/Don’t know |
| If yes, who was diagnosed with cervical cancer? | Friend/Relative/Other – please specify (open response) |
| How likely do you think it is that you will develop cervical cancer? | Very likely/Somewhat likely/Neutral/Somewhat unlikely/Very unlikely |
| Why did you choose your previous answer on about your likelihood of developing cervical cancer? | Open response |
| Imagine that we flip a fair coin 1,000 times. What is your best guess about how many times the coin would come up heads in 1,000 flips? | Open response |
| In the BIG BUCKS LOTTERY, the chances of winning a £10,000 prize is 1%. What is your best guess about how many people would win a £10,000 prize, if 1,000 people each buy a single ticket to BIG BUCKS? | Open response |
| In the ACME PUBLISHING SWEEPSTAKES, the chance of winning a car is 1 in 1,000. What percent of tickets to ACME PUBLISHING SWEEPSTAKES win a car? | Open response |
| Cervical screening prevents as many as 70% of deaths each year in the UK | True/False |
| How certain are you? | Just guessing 50%/ 60%/ 70%/ 80%/ 90%/ Absolutely sure – 100% |
| Cervical screening is mandatory | True/False |
| How certain are you? | Just guessing 50%/ 60%/ 70%/ 80%/ 90%/ Absolutely sure – 100% |
| HPV is a sexually transmitted infection | True/False |
| How certain are you? | Just guessing 50%/ 60%/ 70%/ 80%/ 90%/ Absolutely sure – 100% |
| HPV can be passed on during sexual intercourse | True/False |
| How certain are you? | Just guessing 50%/ 60%/ 70%/ 80%/ 90%/ Absolutely sure – 100% |
| Men can’t get HPV | True/False |
| How certain are you? | Just guessing 50%/ 60%/ 70%/ 80%/ 90%/ Absolutely sure – 100% |
| HPV usually doesn’t need any treatment | True/False |
| How certain are you? | Just guessing 50%/ 60%/ 70%/ 80%/ 90%/ Absolutely sure – 100% |
| The main goal of cervical screening is to find cancer that is already there | True/False |
| How certain are you? | Just guessing 50%/ 60%/ 70%/ 80%/ 90%/ Absolutely sure – 100% |
| An HPV negative test result rules out the possibility that there are any abnormal cells | True/False |
| How certain are you? | Just guessing 50%/ 60%/ 70%/ 80%/ 90%/ Absolutely sure – 100% |
| A women who does not have abnormal cells could get an abnormal test result | True/False |
| How certain are you? | Just guessing 50%/ 60%/ 70%/ 80%/ 90%/ Absolutely sure – 100% |
| If a women is HPV negative but has potentially abnormal cells, she is referred for further testing | True/False |
| How certain are you? | Just guessing 50%/ 60%/ 70%/ 80%/ 90%/ Absolutely sure – 100% |
| You are referred for a colposcopy if you test positive for HPV | True/False |
| How certain are you? | Just guessing 50%/ 60%/ 70%/ 80%/ 90%/ Absolutely sure – 100% |
| All cervical screening samples are tested for abnormal cells | True/False |
| How certain are you? | Just guessing 50%/ 60%/ 70%/ 80%/ 90%/ Absolutely sure – 100% |
| Imagine 1000 women who have attended a cervical screening:  How many of them will have an HPV positive result? | Open response |
| How certain are you? | Just guessing 50%/ 60%/ 70%/ 80%/ 90%/ Absolutely sure – 100% |
| About how many of them could have cells that could be cancer? | Open response |
| How certain are you? | Just guessing 50%/ 60%/ 70%/ 80%/ 90%/ Absolutely sure – 100% |
| All previous screening results are stored on a secure computer system for a minimum of 10 years | True/False |
| How certain are you? | Just guessing 50%/ 60%/ 70%/ 80%/ 90%/ Absolutely sure – 100% |
| A colposcopy checks if there are abnormal cells in the cervix | True/False |
| How certain are you? | Just guessing 50%/ 60%/ 70%/ 80%/ 90%/ Absolutely sure – 100% |
| Imagine a woman has had a cervical screening test. If she gets pregnant later, it is slightly more likely that her baby will be born early due to her having a cervical screening test previously? | True/False |
| How certain are you? | Just guessing 50%/ 60%/ 70%/ 80%/ 90%/ Absolutely sure – 100% |
| Cervical screening can lead to treatment of abnormal cells that is not needed | True/False |
| How certain are you? | Just guessing 50%/ 60%/ 70%/ 80%/ 90%/ Absolutely sure – 100% |
| Cervical screening lowers the risk of getting cervical cancer | True/False |
| How certain are you? | Just guessing 50%/ 60%/ 70%/ 80%/ 90%/ Absolutely sure – 100% |
| Using condoms lowers the risk of getting HPV | True/False |
| How certain are you? | Just guessing 50%/ 60%/ 70%/ 80%/ 90%/ Absolutely sure – 100% |
| The HPV vaccine protects against all types of high-risk HPV which can lead to cervical cancer | True/False |
| How certain are you? | Just guessing 50%/ 60%/ 70%/ 80%/ 90%/ Absolutely sure – 100% |
| After reading all the excerpts from the cervical screening leaflet, how important do you feel it is to attend a cervical screening appointment? | Very important/Somewhat important/Unsure/Somewhat unimportant/Very unimportant |
| Please state your reason why? | Open response |
| Thank you for taking part in this study, if you are interested in taking part within phase B of the study, please leave your email in the box below. | Open response |

**Table 2**: Phase B topic guide

| Interview section | Question | Prompts |
| --- | --- | --- |
| Leaflet utility (pre leaflet interaction) | How do you view the use of an information leaflet as an informative tool? | Usefulness, interaction with the leaflet, age division in informative tools? Tailor further prompts to previous information gathered |
|  | What format for receiving information on cervical screening would you prefer? | Why? Typical for your age group? |
|  | What factors may discourage you from engaging with the leaflet? | Why? |
| Think aloud protocol (for each page) | Information on the page (including numerical information) | Comprehension, interest, helpfulness, likes, dislikes, anything to add/improve or remove/take out of leaflet |
|  | Use of Images on the page | Comprehension, interest, helpfulness, appeal, likes, dislikes, improvements, things to be removed, compliment the written information, do they provide further understanding or not needed, are other images needed to further aid understanding i.e. graphs etc? |
| Leaflet utility (post leaflet interaction) | What is your opinion of the leaflet overall? | Why? |
|  | What is your opinion on the look of the leaflet? | (use of images, graphs, colour, text etc.). Does it appeal to you? |
|  | How confident do you feel about your knowledge on the cervical screening programme/test after reading through the leaflet? | Why? |
|  | Would you use it as your main source of information about the cervical screening procedure? | Why? |
| Barriers and facilitators to leaflet engagement and future screening behaviour | Do you think the information provided within the leaflet is inclusive for everybody who is eligible to attend a cervical screening procedure? | Why? |
|  | To what extent do you feel that a young women’s education level/occupation or income may have impact upon a) how they interpret the leaflet? b) engage with the leaflet c) future screening behaviour? | Why? |
|  | Is the information in the leaflet easy to understand regarding those of a lower education level? | Why? |
|  | Some people have religious or cultural beliefs that might affect whether they take part in cervical screening or how they engage or interpret the leaflet. What are your thoughts about this? | Why? |
|  | To what extent do a women’s/your belief about your risk of developing cervical cancer have upon a) how they/you interpret the leaflet? b) engage with the leaflet c) future screening behaviour? | Why? |
|  | What might people close to you e.g. partner, family, and friends, think about you attending a screening? | Why? |
|  | How do you think the introduction of the HPV vaccine impacts a) how they/you interpret the leaflet? b) engage with the leaflet c) future screening behaviour? Is it beneficial? Is there no need for further screening? (further prompts dependent upon participants response). | Further prompts dependent upon participants response |
|  | If participant had the HPV vaccine: How do you think receiving the HPV vaccine has impacted upon your belief of risk for developing cervical cancer? How has it effected your interpretation/engagement or future screening behaviour? | Why? |
|  | If participant feels HPV vaccine does impact on the screening service :How could the issues that you have identified i.e. less engagement to the screening service due to the vaccine be improved upon? Could the leaflet be changed to encompass these problems? | If yes: how? If no: what do you think the cervical screening programme needs to improve upon this? |
|  | What do you think will help younger people want to engage within the cervical screening program? | Why? |
|  | How important do you feel it is to attend a cervical screening after reading the leaflet? | Why? |
|  | How likely is it that you will attend a cervical screening appointment when the invitation arrives? | Why? |
|  | If already received invite: Although you have already received your invite to the service, how likely is it that you will attend a cervical screening appointment in the near future? | Why? |

**Table 3:** Participant Characteristics

**Survey, Part A (*n* = 120) Interview, Part B (*n* =10)**

***n* (%)** ***n* (%)**

Age (y)

18 2 (1.7) 0 (0.0)

19 5 (4.2) 1 (10.0)

20 6 (5.0) 0 (0.0)

21 9 (7.5) 0 (0.0)

22 29 (24.2) 1 (10.0)

23 34 (28.3) 4 (40.0)

24 35 (29.2) 4 (40.0)

Mean (standard deviation) 22.50 (1.461) 22.90 (1.524)

Education

<GCSE or equivalent 7 (5.8) 0 (0.0)

A level or equivalent 8 (6.7) 2 (20.0)

Higher education or equivalent 105 (87.5) 8 (80.0)

Ethnicity

White 78 (65.0) 9 (90.0)

Mixed – multiple ethic groups 6 (5.0) 0 (0.0)

Asian or Asian British 34 (28.3) 1 (10.0)

Black/African/Caribbean/Black British 1 (0.8) 0 (0.0)

Other ethnic group

Iranian 1 (0.8) 0 (0.0)

Social Grade

A - Higher managerial/professional 24 (20.0) 1 (10.0)

B - Intermediate managerial/professional 23 (19.2) 1 (10.0)

C1 -Supervisory/junior managerial/professional 41 (34.2) 5 (50.0)

C2 - Skilled manual workers 15 (12.5) 2 (20.0)

D - Semi-skilled/unskilled manual workers 7 (5.8) 1 (10.0)

E – Casual/lowest grade workers/unemployed 10 (8.3) 0 (0.0)

First Language

English 80 (66.7) 7 (70.0)

Other 40 (33.3) 3 (30.0)

Numeracy score

1. 14 (11.7) 0 (0.0)
2. 34 (28.3) 2 (20.0)
3. 32 (26.7) 3 (30.0)
4. 40 (33.3) 5 (50.0)

Mean (standard deviation) 1.82 (1.029) 2.30 (0.823)

Religion

No Religion 76 (63.3) 7 (70.0)

Christian – all denominations 23 (19.2) 3 (30.0)

Buddhist 4 (3.3) 0 (0.0)

Hindu 4 (3.3) 0 (0.0)

Muslim 8 (6.7) 0 (0.0)

Sikh 1 (0.8) 0 (0.0)

Other

Christian/Buddhist mix 1 (0.8) 0 (0.0)

Local Religion 1 (0.8) 0 (0.0)

Wiccan 1 (0.8) 0 (0.0)

Not Specified 1 (0.8) 0 (0.0)

Practice Religion (*n* = 44) (Part B *n* = 3)

Yes 25 (56.8) 0 (0.0)

No 19 (43.2) 3 (100.0)

HPV vaccine engagement

Yes 68 (56.7) 7 (70.0)

No 52 (43.3) 3 (30.0)

Perceived cancer risk

Very unlikely 11 (9.2) 1 (10.0)

**Table 3:** Participant Characteristics (continued)

**Survey, Part A (*n* = 120) Interview, Part B (*n* =10)**

***n* (%)** ***n* (%)**

Somewhat unlikely 46 (38.3) 3 (30.0)

Neutral 46 (38.3) 4 (40.0)

Somewhat likely 14 (11.7) 2 (20.0)

Very likely 3 (2.5) 0 (0.0)

Know someone with cervical cancer

Yes 16 (13.3) 1 (10.0)

No 95 (79.2) 9 (90.0)

Don’t know 9 (7.5) 0 (0.0)

Received screening invite (*n* = 119) (Part B *n* = 10)

Yes 19 (15.8) 3 (30.0)

No 100 (83.3) 7 (0.0)

*GCSE – General Certificate of Secondary Education; Variation in sample sizes due to missing data for indicated characteristics, percentages were calculated on the number of participants who answered the question.

**Table 4:** Comparison of Census Data and Participant Characteristics

**Characteristic Census (%) Participants (%)**

Education

<GCSE or equivalent 122,807 (47.8) 7 (5.8)

A level or equivalent 89,566 (34.9) 8 (6.7)

Higher education or equivalent 37,341 (14.5) 105 (87.5)

Ethnicity

White 238,713 (92.3) 78 (65.0)

Mixed – multiple ethic groups 4,712 (1.8) 6 (5.0)

Asian or Asian British 13,435 (5.2) 34 (28.3)

Black/African/Caribbean/Black British 719 (0.2) 1 (0.8)

Other ethnic group 644 (0.2) 1 (0.8)

Religion

No Religion 92,581 (35.8) 76 (63.3)

Christian – all denominations 140,254 (54.2) 23 (19.2)

Buddhist 1,209 (0.4) 4 (3.3)

Hindu 1,239 (0.4) 4 (3.3)

Muslim 6,030 (2.3) 8 (6.7)

Sikh 749 (0.3) 1 (0.8)

Other 646 (0.4) 4 (3.3)

*Figures retrieved from the 2011 Census data (Office for National Statistics, 2011). Please see <https://www.nomisweb.co.uk/census/2011/detailed_characteristics>

**Table 5:** Spearman Rho Correlation Matrix between Interpretation Accuracy, Mean Reported Confidence and Motivation to Engage.

Interpretation Accuracy Reported Confidence (M) Motivation to Engage

Interpretation Accuracy - .437** .097

Reported Confidence (M) - - .229*

Motivation to Engage - - -

** Correlation is significant at the 0.01 level, *Correlation is significant at the 0.05 level.

**Table 6:** Chi Square Analysis measuring the association between HPV vaccine engagement and Perceived cancer risk

HPV Vaccine Engagement Yes No Total

Cancer Risk Perception Very Unlikely 4 7 11

Somewhat Unlikely 28 18 46

Neutral 26 20 46

Somewhat Likely 7 7 14

Very Likely 3 0 3

Total 68 52 120

X^2^(4, N = 120) = 4.72, *P* = .317

**Table 7:** Distribution of Participant Estimates for Item Assessing the Number of Women Expected to have an HPV Positive Result

*Imagine 1000 women who have attended a cervical screening.* *About how many of them will….*

*Have an HPV positive result?*

Correct answer: **130**

Response Frequency (n) Percent (%)

**130 86 71.7**

13 10 8.3

200 3 2.5

300 2 1.7

50 2 1.7

10 1 0.8

120 1 0.8

25 1 0.8

26 1 0.8

4 1 0.8

40 1 0.8

500 1 0.8

80 1 0.8

800 1 0.8

899 1 0.8

8700 1 0.8

90 1 0.8

Other

**Table 8:** Distribution of Participant Estimates for Item Assessing the Number of Women Expected to have Abnormal Cells

*Imagine 1000 women who have attended a cervical screening.* *About how many of them will….*

*Have cells that could be cancer?*

Correct answer: **40**

Response Frequency (n) Percent (%)

**40 71 59.2**

4 9 7.5

10 4 3.3

100 3 2.5

130 3 2.5

20 3 2.5

5 2 1.7

50 2 1.7

15 1 0.8

150 1 0.8

22 1 0.8

30 1 0.8

300 1 0.8

310 1 0.8

400 1 0.8

600 1 0.8

70 1 0.8

800 1 0.8

90 1 0.8

Other 5 5.6
